# Molecular Mechanisms of Manganese Oxide Nanoparticles Toxicity in Brain and Other Tissues: An Overview

**DOI:** 10.31083/FBL47103

**Published:** 2026-05-12

**Authors:** Michael Aschner, Anatoly V. Skalny, Svetlana V. Notova, Margarita N. Tinkova, Rongzhu Lu, Andrey A. Skalny, Abel Santamaria, Ji-Chang Zhou, Alexey A. Tinkov

**Affiliations:** 1 Department of Molecular Pharmacology, Albert Einstein College of Medicine, Bronx, NY 10461, USA; 2 Center of Bioelementology and Human Ecology, Sechenov First Moscow State Medical University, 119435 Moscow, Russia; 3 Department of Medical Elementology, Peoples’ Friendship University of Russia (RUDN University), 117198 Moscow, Russia; 4 Laboratory of Metallomics, Institute of Bioelementology, Orenburg State University, 460018 Orenburg, Russia; 5 Outpatient Department, Orenburg Central District Hospital, 460008 Orenburg, Russia; 6 Department of Preventive Medicine and Public Health Laboratory Sciences, School of Medicine, Jiangsu University, 212013 Zhenjiang, Jiangsu, China; 7 Laboratorio de Nanotecnología y Nanomedicina, Departamento de Atención a la Salud, Universidad Autónoma Metropolitana-Xochimilco, and Facultad de Ciencias, Universidad Nacional Autónoma de México, 04960 Mexico City, Mexico; 8 School of Public Health (Shenzhen), Shenzhen Campus of Sun Yat-sen University, 518107 Shenzhen, Guangdong, China; 9 Laboratory of Ecobiomonitoring and Quality Control, Yaroslavl State University, 150003 Yaroslavl, Russia

**Keywords:** manganese, nanoparticles, brain, toxicity, nanomedicine

## Abstract

The use of manganese oxide nanoparticles (MnO_*x*_NPs) in biomedicine increases the risk of their accumulation in the body, potentially leading to toxicity in various organs and tissues. In addition, occupational exposure to MnOxNPs-containing aerosols may also occur. MnOxNPs have been shown to accumulate in the brain and induce neurobehavioral alterations. However, the specific mechanisms of MnO_*x*_NPs toxicity in the brain and other tissues remain incompletely understood. Therefore, the objective of this review is to summarize existing data on the toxicity of MnO_*x*_NPs in the brain and other tissues, and to discuss the molecular mechanisms underlying their neurotoxic effects. It has been shown that MnO_*x*_NPs induce neuronal death through induction of mitochondrial dysfunction and subsequent apoptosis, and overaccumulation of tau protein and amyloid-*β*. Neurotoxic effects of MnO_*x*_NPs may also be mediated by blood–brain barrier disruption, and dysregulation of dopaminergic and glutaminergic signaling. Exposure to MnO_*x*_NPs induces neuroinflammation through activation of nuclear factor kappa B (NF-*κ*B) and p38 mitogen-activated protein kinase (p38 MAPK) pathways in a reactive oxygen species-dependent manner. *In vitro* studies further demonstrate that MnO_*x*_NPs exhibit a dose-dependent cytotoxic effects in alveolar macrophages, as well as in respiratory, colonic, and other epithelial cells, through the promotion of oxidative stress and an inflammatory response. Overexposure to MnO_*x*_NPs has significant nephrotoxic, hepatotoxic, and immunotoxic effects, as well as affecting the reproductive system. Smaller particles exhibit more pronounced toxic effects in the brain and other tissues than larger nanoparticles or microparticles. However, the mechanisms underlying the different toxicities of MnO_*x*_NPs of different sizes, shapes, and surface modifications remain unclear. These observations highlight the potential of MnO_*x*_NP exposure to contribute to neurological disorders and dysfunction of other systems, underscoring the need for further mechanistic studies to ensure their safe application in biomedicine.

## 1. Introduction

Manganese (Mn) is the third most abundant transition metal in the Earth’s crust [1]. It is also considered an essential metal involved in brain development and functioning, immunity, and metabolic regulation [2]. Mn occurs in the environment in various oxidative states (+2, +3, and +4), giving rise to more than 30 minerals [3], including multiple oxides [4]. Manganese oxides (MnO_*x*_) have been used as pigments in glass and agents in steel production since ancient Egypt and Greece [5]. In the last decades, the investigation and applications of MnO_*x*_ nanoparticles (MnO_*x*_NPs) have been significantly increased [6]. MnO_*x*_NPs are characterized by unique properties including magnetism, semiconductivity, catalytic activity, energy storage, optical properties, biocompatibility, polymorphisms, ion exchange, and phase transformation, which mediate a wide spectrum of applications [7]. Briefly, MnO_*x*_NPs are utilized in water [8] and soil [9] remediation, the optoelectronic and catalytic industries [10], the production of Li-ion batteries [11], and semiconductors [12].

Along with other Mn-containing particles like Mnferrite NPs [13–15], MnO_*x*_NPs are widely used in biomedicine [16]. Briefly, due to the significant magnetization of MnO_*x*_NPs [17], they are used as strong T1 MRI contrast agents [18]. MnO_*x*_NPs also have potential applications in treatment of cancer [19,20] and brain diseases including Parkinson’s disease [21,22], Alzheimer’s disease [23,24], and hypoxic brain damage [25]. In addition, MnO_*x*_NPs are used for drug delivery [26]. Specifically, MnO_2_NPs are loaded with small molecules, peptides, nucleic acids, proteins, as well as phytochemicals [27]. Redox activity of Mn mediates its potential in developing MnO_*x*_-containing nanoconstructs with enzyme-like activity [26]. The use of MnO_*x*_NPs in biomedicine may result their increased body burden, thus raising the issue of their potential toxicity.

Although Mn is an essential metal, it is toxic upon overload, affecting mainly the brain and resulting in neurodegeneration [28]. Congruently, while it is assumed that MnO and MnO_*x*_NPs are characterized by low toxicity due to the essentiality of Mn, several findings showed that these particles possess cytotoxic effects in a number of cell lines [29]. In addition, *in vivo* studies also showed that MnO_*x*_NPs adversely affected the liver and kidneys [30], reproductive system [31], and other systems and organs. Given the neurotoxic effects of Mn, MnO_*x*_NPs may also exert neurotoxicity [32]. Several studies showed that MnO_*x*_NPs impair the viability of brain cells [33,34] as well as affects behavior in laboratory rodents [35,36]. However, the mechanisms of MnO_*x*_NPs toxicity in various organs, and especially the brain, are still disputable.

Therefore, the objective of the present review was to summarize the existing data on the toxicity of MnO_*x*_NPs in the brain and other tissues, as well as discuss the molecular mechanisms underlying their neurotoxic effects.

## 2. Systemic Toxicity of MnO_*x*_NPs

### 2.1 In Vitro Cytotoxicity of MnOxNPs in Non-Neuronal Cells

In view of a wide spectrum of the potential biomedical applications of MnO_*x*_NPs, understanding its adverse health effects is crucial for further clinical applications [7]. Several studies showed that MnO_*x*_NPs possess cytotoxic effects in various types of cells which may be mediated by ROS overproduction. Specifically, *in vitro* data showed that Mn_3_O_4_NPs induce an increase in intracellular ROS production [37]. Mn_2_O_3_NPs also possess a high capacity for cytochrome c oxidation, exceeding that observed for other nanoparticles, including CeO_2_, TiO_2_, BaSO_4_, carbon black, Cu, Ag, and ZnO NPs. This oxidative potential was mediated by reduced glutathione (GSH) and ascorbic acid depletion [38]. Prooxidant effect of Mn_2_O_3_NPs characterized by hydroxyl radical generation was also revealed in an assay of L-3,4-dihydroxyphenylalanine (L-dopa) autoxidation [39]. It has also been shown that ability of MnO_2_NPs to generate ROS through Fenton-like mechanism and oxidize cytochrome c is not associated with Mn^2+^ dissolution, which was insignificant [40]. Finally, prooxidant activity of Mn-containing welding fume nanoparticles from stainless steel directly correlated with Mn^2+^ release from the particles [41]. Congruently, commercially available MnO_*x*_NPs of various shapes (spherical, rods, flakes) mainly sized around 30 nm induced a significant decrease in viability of A549, HepG2, and J774A.1 cells due to ROS overproduction and caspase 3 activation [29].

Significant cytotoxic effects of MnO_*x*_NPs were revealed in cultures of airway epithelial cells. It has been demonstrated that Mn_2_O_3_NPs exposure decreases viability of both BEAS-2B and A549 cells through induction of apoptosis [42]. In BEAS-2B cells and RAW 264.7 macrophages, exposure to Mn_2_O_3_ resulted in a dose-dependent decline in cell viability, energy production, and membrane leakage. Further analysis also showed that along with membrane damage Mn_2_O_3_ treatment induced mitochondrial dysfunction with mitoROS production [43]. Although Mn_3_O_4_NPs did not possess cytotoxicity upon short-term exposure (24 h), long-term exposure (9 days) was associated with significant cytotoxicity in A549 and especially Caco-2 cells [44]. It has also been demonstrated that exposure to Mn_3_O_4_NPs induces ROS overproduction in airway epithelial A549 cells and colorectal adenocarcinoma HT29 cells, although this prooxidant effect was associated with reduced cell viability only in A549 cells. Furthermore, exposure to a novel nanomaterial, GNA35, based on Mn_3_O_4_NPs with enhanced electrochemical properties, induced a more severe prooxidant effect and decrease in A549 cells viability [45]. It has been also demonstrated that Mn_2_O_3_NPs exposure significantly reduces A549 viability through induction of apoptosis and slightly inhibits cell proliferation [46]. Another study in a culture of A549 cells demonstrated that exposure to Mn oxide nanoparticles increases ROS production, up-regulates transferrin, ferritin light and heavy chains, and DMT1 mRNA expression, as well as increases IL-6 and IL-8 mRNA and protein expression, although some of these effects were more profound upon exposure to Fe oxide NPs or mixed type NPs [47]. Finally, it has been demosntrated that IC_50_ of Mn_3_O_4_NPs accounts for 98 μg/mL [48]. In another lung cell line (FE-1) MnO_2_NPs dose-dependently induced DNA damage, exceeding that observed in MnO_2_MPs-exposed cells [49]. Comparative analysis of Mn_3_O_4_NPs and MnSO_4_ showed that, despite both forms of Mn induce caspase-3-mediated apoptosis in CCL-149 alveolar epithelial cells, only Mn_3_O_4_NPs promoted ROS production and GSH oxidation. Noteworthy, exposure to MnNPs resulted in higher intracellular Mn content compared to MnSO_4_ [50]. Another study also showed that Mn_3_O_4_NPs increase ROS production in CCL-149 airway epithelial cells, although not reducing the metabolic activity of cells [51]. Congruently, Mn_2_O_3_NPs induced a significant decline in human bronchial epithelial NCI-292 cell viability with up-regulation of IL-8 mRNA and protein expression. At the same time, development of oxidative stress upon exposure to Mn_2_O_3_NPs induced up-regulation of SOD2 and HO1 mRNA expression in NCI-292 cells [38]. In another line of human bronchial epithelial cells (16HBE14o-), exposure to Mn_2_O_3_ resulted in a dose dependent decrease in cell metabolic activity assayed by MTT test with IC_50_ of 33 mg/L [52].

Adverse effects of MnO_*x*_NPs exposure were also revealed in alveolar macrophages. Specifically, exposure to Mn_3_O_4_NPs induced a significant increase in ROS production in CRL-2192 alveolar macrophages, resulting in a significant reduction in cellular metabolic activity and down-regulation of MCP-1 production. Increased caspase-3 activity in Mn_3_O_4_NPs-exposed cells is indicative of apoptosis. Attenuation of these effects by Trolox treatment showed that cytotoxicity of Mn_3_O_4_NPs in alveolar macrophages is ROS-dependent [51]. Correspondingly, MnO_2_ NPs reduced the viability of RAW264.7 macrophages, also decreasing their phagocytic activity due to the enhancement of TNF*α* and down-regulation of IL-1*β* mRNA expression. These effects were reversed by doping MnO_2_NPs with ZnO, which also resulted in a more profound increase in ROS production and promoted antioxidant response through up-regulation of Nrf2 protein expression [53].

Certain studies showed that MnO_*x*_NPs are cytotoxic to intestinal epithelial cells *in vitro*. It has been also demosntrated that Caco-2 cells (IC50 = 66.7 μg/mL) were more sensitive than lung epithelial A549 cells (IC50 = 136.2 μg/mL) and mouse Balb/c 3T3 fibroblasts (IC50 = 195.0 μg/mL) to Mn_3_O_4_NPs-induced cytotoxicity. The latter was shown to be dependent on ROS generation, but not metal ion release [54]. In a culture of Caco-2 cells exposure to various MnOxNPs resulted in a dose-dependent reduction in cell viability with cytotoxicity decreasing in the following order: Mn_3_O_4_ > Mn_3_O_2_ > MnO_2_. However, in presence of H_2_O_2_, non-cytotoxic doses of MnO_*x*_NPs increased cell viability, being indicative of both prooxidant and antioxidant effects of MnO_*x*_NPs. Specifically, MnOxNPs possessed ascorbate (Mn_3_O_2_ > MnO_2_ > Mn_3_O_4_) and GSH-oxidizing (Mn_3_O_4_ > Mn_3_O_2_ > MnO_2_) activities, while promoting H_2_O_2_ decomposition (MnO_2_ > Mn_2_O_3_ > Mn_3_O_4_) [55]. These findings are generally in agreement with earlier indications of the protective effects of Mn_3_O_4_NPs against H_2_O_2_-induced cytotoxicity in murine insulinoma *β*TC3 cells by mimicking antioxidant enzyme activity, although at high doses the particles induced a significant decrease in cell viability [56].

Cytotoxic effects of MnO_*x*_NPs were also evident in other epithelial cell lines. Exposure of epithelial MCF-7 and HT1080 cells to MnO_2_NPs resulted in increased total ROS and mitoROS production, inhibition of SOD, CAT, and glutathione reductase (GR) activity, and depletion of GSH pool. Along with induction of oxidative stress, activation of proapoptotic signaling by up-regulation of p53 and Bax mRNA expression, with repression of Bcl-2 mRNA, and reduction of mitochondrial membrane potential result in a decrease in cell viability. However, the results show that the effects of MnO_2_NPs were more profound in HT1080 cells compared to MCF-7 cells [57]. In human cervical carcinoma cells (HeLa) and mouse fibroblast cells (L929), exposure to silica-coated MnONPs induced a significant increase in ROS production, mitochondrial dysfunction, and G2/M phase arrest. Along with the up-regulation of p53 protein expression and the reduction of Bcl-2/Bax ratio, these effects were associated with caspase-3 activation, leading to cell apoptosis [58].

Finally, Mn_3_O_4_NPs exposure significantly reduced viability of mouse embryonic stem (mES) cells with up-regulation of gene products associated with oxidative stress (Srxn1), DNA damage (Rtkn), endoplasmic reticulum stress (Ddit3) and p53-related stress (Btg2), although this effect was less pronounced than in the case of metallic MnNPs [59].

However, it is demosntrated that synthesis of NPs also significantly affects particle toxicity. In hMSC cells chemically synthesized MnO_2_NPs induced significant alterations in cell volume and shape, while green-synthesized NPs did not possess adverse effects on cell morphology, thus showing its lower cytotoxicity [60].

Generally, these findings show that MnO_*x*_NPs possess cytotoxicity in airway epithelial cells, alveolar macrophages, enterocytes, and other epithelial cells (Table 1, Ref. [38,42–46,48–55,57–59]). Evidence shows that the cytotoxicity of MnO_*x*_NPs is mainly mediated by their prooxidant activity. Specifically, overproduction of ROS upon MnO_*x*_NPs exposure, along with induction of mitochondrial dysfunction, results induces cell cycle arrest and apoptosis, resulting in reduced cell viability. In addition, MnO_*x*_NPs-induced oxidative stress is associated with up-regulation of proinflammatory cytokine expression, which also contributes to cytotoxicity.

### 2.2 Systemic toxicity of MnOxNPS In Vivo

Agreeing with cellular models, *in vitro* studies also show potential toxicity of MnO_*x*_NPs exposure for various organs. Specifically, exposure to MnNPs via intraperitoneal injection in rats induced histopathological damage, including hepatocyte degeneration, central vein congestion, sinusoid dilatation, and hemorrhages in the liver, capsule rupture, inflammatory infiltration, and glomerulosclerosis in the kidneys, and epithelial desquamation, impaired tubular morphology, and vacuoles in the testis [31]. Correspondingly, intraperitoneally injected Mn_3_O_4_NPs induced severe liver injury characterized by hepatic inflammation and cell exfoliation along with induction of hepatocyte apoptosis through up-regulation of caspase-3 protein expression and Bax/Bcl2 ratio. In addition, Mn_3_O_4_NPs exposure up-regulated expression of genes involved in endoplasmic reticulum stress (*GRP78*, *Climp63*) xenobiotic metabolism by cytochrome P450 (*CYP1A2*, *Sult2a1*). The latter may contribute to ROS overproduction, playing a key role in hepatocyte apoptosis, as evident from inhibition of apoptosis upon treatment with antioxidant NAC [61]. In yellow catfish, Pelteobagrus fulvidraco, exposure to MnO_2_NPs induced a more profound toxic effect in liver characterized by up-regulation of pro-inflammatory (*il1β, il8, tnfa*) and pro-apoptotic (*bax, casp3, cycs*) gene expression compared to MnSO_4_. Furthermore, liver damage is also associated with lipid accumulation due to activation of lipogenic *fas, acsl3, scd1, gpat3, agpat2* and *dgat1* mRNA expression. It is assumed that activation of hepatic lipogenesis upon exposure to MnO_2_NPs may be mediated by down-regulation of miR-92a and miR-92b-3p expression, which inhibit lipid accumulation by direct targeting *acsl3* [62]. Another epigenetic mechanism of MnO_2_NPs-induced hepatic lipogenesis in yellow catfish may include down-regulation of miR-20a-5p which targets mitofusin 2 (*mfn2* gene), promoting mitochondrial fusion and interaction between mitochondria and lipid droplets together with Plin2 [63]. Further studies showed that MnO_2_NPs exposure induce mitoROS overproduction leading to up-regulation of heat shock factor 1 (Hsf1) mRNA and protein expression, as well as promotion of its phosphorylation at Ser326 residue. The latter promotes Hsf1 nuclear translocation and its binding to *dgat1*, *plin2* and *bnip3* promoters, resulting in activation of lipogenesis and mitophagy, as observed in MnO_2_NPs-treated hepatocytes [64]. Finally, a study in broilers showed that Mn_2_O_3_NPs-induced liver damage characterized by apoptosis, mitophagy, and altered mitochondrial dynamics is associated with intestinal inflammation and reduced gut wall integrity due to down-regulation of claudin1, occludin, MUC1 and ZO-1 mRNA and protein expression. Furthermore, intestinal damage was also accompanied by gut dysbiosis characterized by increased *Firmicutes*/*Bacteroidetes* ratio, and reduced relative abundance of *Bacteroides* and *Bifidobacterium*, along with enrichment of *Pseudomonas*, *Ralstonia*, *Prauserell*, *Sedimini-bacterium*, *Burkholderia*-*Caballeronia*, *Paraburkholderia*, *Bradyrhizobium* and *Rubrobacter* [65]. Another study demonstrated that intraperitoneal injection of MnO_2_NPs possesses hepatotoxicity and nephrotoxicity, evidenced by elevated ALT, AST, and ALP activity, increased blood urea nitrogen, creatinine, and total bilirubin concentrations, along with induction of hyperglycemia. Noteworthy, all adverse effects of MnO_2_NPs were attenuated by vitamin D administration [30]. Correspondingly, subcutaneous injection with MnO_2_NPs and MPs in rats resulted in a significant increase in blood glucose and cholesterol levels. Furthermore, exposure to both types of particles resulted in a significant reduction in HDL-C level without any significant effect on LDL-C and TG concentrations [66].

In addition, intravenous injection with Mn_3_O_4_NPs (via tail vein) weekly for 120 days resulted in accumulation of Mn in testes and alteration of testicular morphology characterized by reduced thickness of the germinative layer and degeneration of seminiferous tubules. These effects were associated with reduced levels of testosterone and FSH, as well as decreased sperm count in ejaculate, reduced sperm motility, and a higher rate of abnormalities. Adverse reproductive effects of Mn_3_O_4_NPs exposure may be mediated by overproduction of ROS, induction of mitochondrial dysfunction, and subsequent apoptosis, as observed in a culture of TM4 cells. Furthermore, transcriptional analysis of testes revealed up-regulation of genes involved in PPAR-signaling (Fabp1, Apoa2, Apoa3, and Pck1), steroid hormone synthesis (LOC100361547, Cyp2c12, Cyp2c6v1, and Ugt2b37), and cytochrome P450-mediated metabolism (Gsta4, LOC102550391, Ugt2b37, Sult2a2) [67]. Another study showed that MnO_2_NPs also induce testicular damage characterized by a decline in the number of spermatogonial cells, primary spermatocytes, spermatids, and Leydig cells, resulting in a reduction sperm number, motility, and viability, along with increased sperm abnormalities, and altered reproductive hormone levels. The latter may be mediated by MnO_2_NPs-induced down-regulated mRNA expression of StAR, HSD-3*β*, CYP11A1 involved in steroidogenesis [68]. Oral exposure to Mn_2_O_3_NPs also significantly reduced serum luteinizing hormone (LH), follicle-stimulating hormone (FSH), and testosterone levels, as well as resulted in a significant decrease in the number of spermatogonial cells, primary spermatocytes, spermatids, and Leydig cells in testes. Interstitial edema of seminiferous tubules and vacuolar degeneration of epithelium was also observed [69]. Finally, subcutaneous injection of MnO_2_NPs induced a decline in the number of sperms, spermatogonia and spermatocytes, decreased sperm motility, and reduced diameter of seminiferous tubes, although this effect was more profound upon exposure to MnO_2_ microparticles [70].

Intratracheal instillation of Mn_3_O_4_NPs in a dose of 0.25 mg per animal resulted in a significant increase in the number of neutrophils and increased neutrophil-to-alveolar macrophage ratio in BALF, as well as increased amylase and LDH activity, altogether indicating pulmonary inflammation and toxicity [71]. Correspondingly, exposure of CB57BL/6 mice to Mn_2_O_3_NPs significantly increased BALF neutrophil count, as well as MCP-1 and IL-6 levels [43].

Certain studies show that MnO_*x*_NPs exposure may adversely affect other organs and tissues. Specifically, oral exposure to 125 mg/kg and 250 mg/kg BW Mn_3_O_4_NPs for 20 days did not induce histopathological effects in the liver, spleen, kidneys, or colon, while inducing an inflammatory response in the colon by up-regulating IL-1*β* and IL-6 levels. Down-regulation of tight junction protein claudin-1 in the colon induced by exposure to high-dose Mn_3_O_4_NPs was associated with gut dysbiosis. Specifically, high-dose exposure significantly reduced gut biodiversity and the total number of microbial species. At the phylum level, the relative abundance of *Verrucomicrobiota*, *Bacteroidota*, and *Firmicutes*_C was reduced in response to both low and high doses of Mn_3_O_4_NPs [72]. In rat erythrocytes, Mn_3_O_4_NPs promoted eryptosis due to an increase in cellular Ca^2+^ level, ROS and RNS overproduction, phosphatidylserine externalization, calpain and caspases 3 and 9 activation [73]. Noteworthy, replacement of MnCO_3_ in diet with Mn_2_O_3_NPs maintained Mn balance in rats, although resulting in impaired femoral bone morphology characterized by bone fibrosis and steatosis [74]. Studies in avian species show that MnO_*x*_NPs may also induce significant immunotoxic effects. It has been demonstrated that dietary exposure to Mn oxide NPs in broilers impairs immune response, evidenced by lower concentration of antibodies against Newcastle disease and infectious bronchitis [75]. Correspondingly, administration of Mn_2_O_3_NPs induced more caspase 3 activation in blood immune cells of turkeys compared to bulk MnO [76].

Taken together, the increasing body of evidence shows that exposure to MnO_*x*_NPs possesses reproductive toxicity, induces hepatic and kidney damage, promotes intestinal inflammation, as well as induces immunotoxicity (Table 2, Ref. [30,43,61–72,74]). In contrast, smartly engineered casein MnO_*x*_NPs generated with MnCl_2_ and casein under alkaline conditions [77] did not induce toxic effects upon both acute and chronic exposures [78]. These findings show that modified MnO_*x*_NPs are characterized by lower toxicity, while unmodified particles possess cytotoxic effects, and thus dissolution of MnO_*x*_NPs from modified particles must be limited [26]. These findings are in agreement with the role of metal nanoparticle surface coating in reducing its toxicity and facilitating their therapeutic effect in treatment of brain diseases [79].

## 3. Neurotoxicity of MnO_*x*_NPs

Given the role of Mn^2+^ release from MnO_*x*_NPs as one of the mechanisms of its biological activity [80], as well as neurotoxic effects of Mn^2+^ [28], it has been assumed that exposure and brain accumulation of MnO_*x*_NPs results in brain damage [32].

### 3.1 Toxic Effects of MnOxNPs in Brain Cells In Vitro

Mn_3_O_4_NPs induce cell death in dopaminergic PC12 cells by induction of oxidative stress and apoptosis through up-regulation of mitochondrial calcium (Ca^2+^) uniporter (MCU) and subsequent increase in mitochondrial Ca^2+^ concentration. The role of Ca overload in cytotoxic effects of Mn_3_O_4_NPs in PC12 cells was also confirmed by the observed inhibition of apoptosis and oxidative stress by treatment with the MCU inhibitor. Furthermore, a decrease in dopamine content in Mn_3_O_4_NPs-exposed cells and rats was mediated by downregulation of DOPA decarboxylase (DDC) expression [33]. Correspondingly, MnNPs effectively internalized by PC12 cells induce a significant decrease in cellular dopamine, DOPAC, and HVA contents, and this effect was similar to that observed upon exposure to soluble Mn^2+^ (Mn acetate). Although exposure to MnNPs induced only a slight mitochondrial dysfunction, it resulted in a more than 10-fold increase in ROS production, exceeding the respective values observed for Mn^2+^ [34]. In addition, a comparative study showed that Mn_2_O_3_NPs resulted in more profound decrease in PC12 cell viability compared to Mn_3_O_4_NPs and especially Mn_5_O_8_NPs. This effect was associated with a species-specific decrease in cellular GSH levels by Mn_2_O_3_NPs followed by Mn_3_O_4_NPs and Mn_5_O_8_NPs [81].

In another model of dopaminergic neurons, N27 cells, exposure to MnNPs also induced cell death associated with ROS overproduction and caspase-3-mediated proteolytic cleavage and activation of protein kinase C*δ* (PKC*δ*), although treatment with antioxidants did not alleviate MnNPs-induced neuronal toxicity. In addition, MnNPs induced autophagy in N27 cells evidenced by Beclin1 cleavage and an increase in LC3I/LC3II ratio. Neurotoxic effect of MnNPs was also observed in primary dopaminergic neurons, being associated with reduced neurite length [82]. Concomitantly, another study showed that a decrease in PC12 cell dopamine content induced by exposure to 10 μg/mL MnNPs failed to alter the expression of genes involved in redox homeostasis (thioredoxin reductase 1 (*Txnrd1*), glutathione synthetase (*Gss*), and glutathione peroxidase 1 (*Gpx1*)) or dopamine metabolism (tyrosine hydroxylase (*Th*), monoamine oxidase A (*MaoA*), and catechol-O-methyltransferase (*Comt*), vesicular monoamine transporter-2 (*Vmat-2*), and dopamine transporter (*Dat*)), being related to up-regulation of *α*-synuclein and down-regulation of Parkin gene expression [83].

Exposure of neuronal SH-SY5Y cells to MnNPs led to GSH depletion and a significant increase in cellular ROS production due to mitochondrial dysfunction, altogether resulting in oxidative DNA damage, chromosome fragmentation, phosphatidylserine translocation, and caspase-3 activation, contributing to cellular apoptosis [84]. Correspondingly, Mn_2_O_3_NPs exposure induced both necrosis and apoptosis in a dose-dependent manner in SH-SY5Y neuroblastoma cells. The latter was mediated by caspase-3 and caspase-9 activation and increased Bax/Bcl-2 ratio. In addition, molecular docking and molecular dynamic studies showed that Mn_2_O_3_NPs can bind tau protein and promote its folding, potentially contributing to neurotoxic effects [85]. Mn_3_O_4_NPs, especially small-sized, were shown to have more profound effects in dividing ST-14 striatal neuroblasts due to induction of mitochondrial dysfunction and ROS production with subsequent increase in NF-*κ*B transcription [86].

Cellular studies also show that MnO_*x*_NPs contribute to neuroinflammation. Specifically, in BV2 microglial cells MnO_2_NPs exposure resulted in a significant increase in ROS production and p38 MAPK pathway activation, ultimately leading to overexpression of IL-1*β* and TNF*α*. In turn, treatment with antioxidant N-acetylcysteine and p38 MAPK inhibitor (SB203580) abrogated secretion of proinflammatory cytokines in microglia, indicative of the key role of ROS/p38 MAPK pathway in neuroinflammation induced by MnO_2_NPs exposure [87]. Correspondingly, in a co-culture of neuronal CATH.a cells and C8-B4 microglia, exposure to MnNPs induced a significant decrease in cell viability, ROS overproduction, and up-regulation of *Tnf-α* and *Il-6* gene expression. Next-generation sequencing (NGS) also revealed that proinflammatory effects of MnNPs exposure were associated with modulation of expression of miR-124–3p, miR-1–3p, miR-16–5p, let-7a-5p, and miR-155–5p, considered as upstream regulators. Furthermore, up-regulation of miR-155–5p abrogated the stimulatory effects of MnNPs exposure on TNF*α* and IL-6 mRNA and protein expression in a co-culture. These findings demonstrate that induction of neuroinflammation by MnNPs may be at least partially mediated by modulation of miR-155–5p expression [88].

**Fig. 1.**
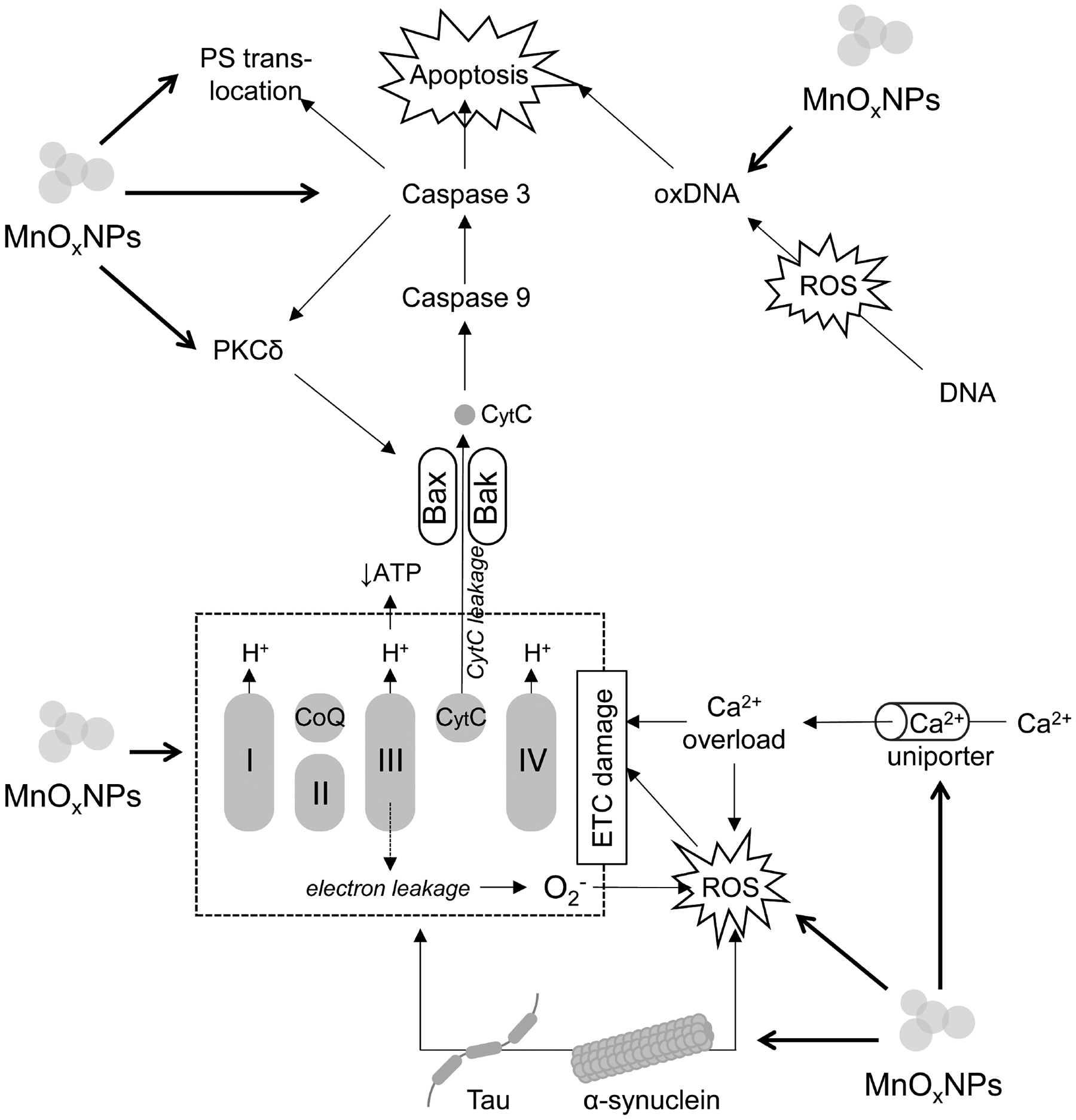
The molecular mechanisms of MnO_*x*_NPs-induced neuronal death. Briefly, MnO_*x*_NPs exposure inhibits electron transport chain (ETC) functioning resulting in reduction of ATP production and increased electron leakage at complexes I and III with subsequent formation of ROS. In addition to ROS formation, MnO_*x*_NPs increase activity of Ca^2+^ uniporter resulting in mitochondrial Ca^2+^ overload. Both ROS overproduction and Ca^2+^ overload further affect ETC functioning. MnO_*x*_NPs induce tau and *α*-synuclein formation, which promote oxidative stress, Ca^2+^ accumulation, and ETC damage. MnO_*x*_NPs -induced mitochondrial dysfunction results in increased cytochrome c leakage with subsequent activation of caspases 9 and 3, leading to apoptosis. Caspase 3 also mediates proteolytic cleavage and activation of PKC*δ*, which up-regulates Bax further promoting cytochrome c release. In addition, caspase 3 activation may be also responsible for phosphatidylserine (PS) translocation to the outer surface of the cell membrane, promoting phagocytosis of apoptotic cell. Furthermore, MnO_*x*_NPs -induced oxidative stress results in DNA oxidation with accumulation of oxidized DNA (oxDNA), which also triggers apoptosis. Thin arrows are indicative of the continuum of changes, thick arrows show stimulatory effect of MnO_*x*_NPs on these mechanisms. MnO_*x*_NPs, MnO_*x*_ nanoparticles; ATP, adenosine triphosphate; ROS, reactive oxygen species; PKC*δ*, protein kinase C*δ*.

Taken together, the results of *in vitro* studies highlight cytotoxic effects of pristine MnO_*x*_NPs in both neuronal and non-neuronal brain cells (Table 3, Ref. [33,34,82–88]). The existing data show that MnO_*x*_NPs induce neuronal death through a variety of mechanisms including oxidative stress, mitochondrial dysfunction, altered Ca^2+^ homeostasis, induction of tau and A*β* accumulation, and apoptosis (Fig. 1). In addition, MnO_*x*_NPs-induced microglia activation with up-regulation of proinflammatory cytokine expression due to activation of ROS/p38 MAPK pathway and modulation of microRNA expression may also contribute to brain damage through induction of neuroinflammation.

### 3.2 Neurotoxic Effects of MnOxNPs In Vivo

Findings from animal models generally corroborate the results of *in vitro* studies. Intratracheal exposure to MnO_2_NPs results in a more than 5-fold increase in Mn content in brain, whereas the level of Mn in the liver is elevated by less than 2-fold, indicative of the role of the brain as a target for Mn accumulation following MnNPs exposure [89]. An *in vivo* study demonstrated that intratracheal instillation with MnO_2_NPs resulted in a significant increase in both blood and brain Mn accumulation after 9 weeks of exposure [90,91].

Elder *et al*. (2006) [92] demonstrated that the olfactory neuronal pathway is the predominant mechanism of transport of MnNPs into the brain upon inhalation. Specifically, the authors observed a more profound increase in the olfactory bulb Mn content (3.5-fold) compared to lungs (2-fold) upon exposure to MnNPs. Furthermore, in the exposed animals, up-regulation of TNF*α*, MIP-2, GFAP, and neuronal cell adhesion molecule (NCAM) gene and/or protein expression in the olfactory bulb was more profound compared to other brain areas, while no inflammatory effects were observed in lungs [92]. Correspondingly, Romashchenko *et al*. (2019) [93] revealed a significant role of olfactory transport in MnNPs transport into the brain, being more effective than permeation of NPs through BBB [93].

Additional studies showed that exposure to MnO_*x*_NPs results in significant alteration in brain functioning. Specifically, intratracheal instillation with MnO_2_NPs significantly reduced ambulation and rearing with increased local activity and immobility due to increased frequencies of spontaneous cortical activity, elongation of cortical evoked potential latency, as well as decreased nerve conduction velocity [90,91]. This effect was dependent on the size of the exposing particles. It has been shown that alterations in spontaneous cortical activity were more profound upon exposure to smaller MnO_2_NPs (9 and 42 nm) compared to larger (118 nm) particles [94]. Furthermore, the changes in spontaneous and evoked cortical activity and peripheral nerve action potential positively correlated not only with cortical Mn^2+^ content, but also cortical TBARS level, being indicative of the role of lipid peroxidation in MnO_2_NPs-induced neuro-functional alterations [95]. When compared to the effects of soluble MnCl_2_, MnNPs exposure exerted less profound effects on the latency of cortical evoked potentials and tail nerve conduction velocity [96]. However, combined exposure of MnCl_2_ (orally) and MnNPs (intratracheally) for 3 weeks possessed more profound effects on cortical sensory evoked potential and nerve action potential compared to 6-week exposure to MnCl_2_ at the same cumulative dose [97].

MnNPs-induced alterations in evoked potential latency and nerve conduction velocity were abolished by antioxidant-rich green tea, whereas other adverse effects of MnNPs exposure, like reduced body weight gain, were not reversed by green tea administration [95]. Respectively, administration of antioxidants attenuated adverse effects of MnNPs, with rutin being the most effective, whereas ascorbic acid only abolished alterations in cortical evoked potentials, and curcumin did not exert any protective effects [98]. Intraperitoneal injection of Mn_3_O_4_NPs resulted in a significant accumulation of Mn in brain, and dose-dependent reduction in the number of head-dips into holes, brain weight as well as an increase in the number of cells without a nucleolus in caudate nucleus and hippocampus (CA1) [99], which were completely abrogated by administration of a complex of antioxidants (N-acetylcysteine, vitamins A, C, E, selenium) and other bioactive compounds, including pectin, glutamate, glycine, iodide, and omega-3 polyunsaturated fatty acids [100]. These findings show that the neurotoxic effects of MnNPs depend on the prooxidant effect of the particles, although the different efficiencies of various antioxidants may be indicative of the contribution of various sources of ROS in MnNPs neurotoxicity.

Mitochondrial dysfunction is a primary source of ROS upon MnO_*x*_NPs exposure. Specifically, Mn particles induced a significant increase in mitochondrial ROS production at respiratory complexes I and III, being more profound in MnO_2_NP-exposed mitochondria compared to MnO_2_MPs. Furthermore, exposure to MnO_2_NPs, but not MnO_2_MPs, possessed inhibitory effects on complex II and IV activity in the brain [101].

Acute exposure to MnNPs induced significant neurotoxic effects characterized by depression and loss of reaction to auditory stimuli [102], which were associated with severe cerebellar damage characterized by neuronal and glial cell shrinkage and pyknosis, ischemia, and edema [103]. In addition, the route of MnOxNP exposure induces distinct changes in brain pathomorphology. Specifically, acute inhalation exposure to MnO_2_NPs (15–29 nm) in rats resulted in brain and especially cerebellum damage, characterized by ischemic damage to neurons and microglia, endothelial swelling in cerebral blood vessels, and neurite defects. In turn, acute oral exposure resulted in brain edema, vascular hyperemia in the brain cortex and cerebellum, neurite demyelination, as well as alterations in the granular cortex layers [104].

Exposure of adult male Wistar rats to 50–100 μg/kg MnO_2_NPs through intraperitoneal injection resulted in a significant increase in immobility time and reduced sucrose preference. Further analysis showed that MnO_2_NPs induced a significant increase in hippocampal ROS production and lipid peroxidation. These effects were also associated with neuronal apoptosis and necrosis in the hippocampus, as well as a dose-dependent decrease in hippocampal catecholamine content [105]. Surprisingly, adverse neurophysiological effects of MnNPs were attenuated when coexposed to Fe_2_O_3_NPs, while this effect was independent of brain Mn accumulation [106].

Further, it has been shown that alterations in sensory motor functions induced by intraperitoneal MnO_2_NPs exposure in rats were associated with BBB damage with the resulting brain edema and reductions in blood flow in the sensory-motor cortex, hippocampus, caudate putamen, cerebellum, and thalamus, and to a lesser extent in hypothalamus, pons, medulla, and spinal cord. In addition, a dose-dependent increase in neuronal damage characterized by vacuolation, chromatolysis, and Nissl substance loss was observed predominantly in parietal and temporal cortex, hypothalamus, and thalamus, followed by other brain areas [35]. In addition, MnO_2_NPs-induced alterations in learning and memory are associated with hippocampal lesions characterized by edematous cells with karyopyknosis and irregular arrangement, cell connections loss, increased number of irregular cells, as well as damage to the choroid plexus, a blood–cerebrospinal fluid barrier structure. Transcriptomic analysis of the choroid plexus showed that MnNPs exposure affects expression of transporter, ion channel proteins, and ribosomal genes, including BMP/Retinoic Acid Inducible Neural Specific 1 (*Brinp*), synaptoporin (Synpr) and Collapsin response mediator protein 1 (Crmp1) [36].

It has also been demonstrated that MnO_2_NPs exposure induces neuroinflammation due to astrocyte activation, evidenced by increased glial fibrillary acidic protein (GFAP) immunoreactivity, which was more profound in cerebellum, hippocampus, and thalamus [35]. Induction of astrocyte activation and neuroinflammation with the increased number of GFAP and iNOS-positive cells upon injection of MnO_2_NPs to the substantia nigra was also observed [107]. Furthermore, replacement of Mn^2+^ (as MnCO_3_) in the diet with Mn_2_O_3_NPs induced neuroinflammation, evidenced by a significant increase in brain TNF*α* level and ceruloplasmin activity, while reducing IgG levels. At the same time, replacing Mn^2+^ with Mn_2_O_3_NPs did not induce proinflammatory effects, neither in the jejunum nor at the systemic level, indicating higher sensitivity of the brain to the proinflammatory effect of MnNPs [108]. Intragastric administration of MnONPs resulted in severe lymphoid infiltration of brain tissue, while MnOMPs possessed lower toxicity [109].

Several studies show that MnO_*x*_NPs impair neurotransmitter metabolism *in vivo*. Specifically, injection of MnO_2_NPs to the substantia nigra induced a significant decrease in the number of TH-positive neurons, which was similar to that observed upon exposure to dopaminergic neurotoxin 6-hydroxydopamine (6-OHDA) [107], indicating altered dopaminergic neurotransmission. Metabolomic analysis of brain tissues of rats intravenously injected with MnNPs showed that, irrespective of the dose and period of exposure, MnNPs exposure altered brain guanosine, phenylalanine, GABA, myo-inositol, uracil, and propionate levels. Upon short-term exposure, MnNPs at both doses also increased brain glutamate content. These findings demonstrate that exposure to MnNPs altered the glutamine–glutamate/GABA cycle in the brain [110]. Replacement of dietary MnCO3 with the respective dose of Mn2O3NPs resulted in a significant reduction in brain 5-HT levels along with an increase in norepinephrine and dopamine levels, which may be associated with gut microbiota alterations. The latter was characterized by reduced intestinal levels of SCFA, including acetate, propionate, butyrate, decreased enzymatic activity of caecal bacteria, as well as elevated ammonia concentrations and increased pH in caecum [111]. MnO_2_NPs-induced Mn accumulation in the brain was also associated with down-regulation of brain AChE, Na/K-ATPase, Mg^2+^-ATPase, and Ca^2+^-ATPase activities, as well as neuronal vacuolation and inflammatory cell infiltration [112].

Noteworthy, a study in iridescent shark (*Pangasianodon hypophthalmus*) demonstrated that MnNPs exposure resulted in a significant down-regulation of acetyl choline esterase (AChE) activity in the brain, accompanied by significant up-regulation of brain catalase, glutathione-S-transferase, and glutathione peroxidase activity with a concomitant decrease in SOD activity. Furthermore, a dose-dependent increase in hepatic cortisol level may also be indicative of altered hypothalamus-pituitary-inter-renal axis activity. Noteworthy, these effects of MnNPs were nearly similar to those of Mn^2+^, although the latter possessed higher toxicity (LC_50_= 111.75 mg/L) compared to MnNPs (LC_50_ = 93.81 mg/L) [113]. These findings assert the role of Mn^2+^ release in mediating the toxic effects of MnNPs. This is corroborated by the observed formation of stress-granules through eIF2a phosphorylation and inhibition of oxidative phosphorylation in human glioblastoma U87 MG cells, which were also induced by Mn^2+^ exposure [114].

Taken together, the existing data show that MnO_*x*_NPs exposure induces neurotoxicity through a variety of mechanisms (Table 4, Ref. [35,36,89,94–96,99,101,102,105, 107,108,110–112]). Along with induction of ROS production with subsequent development of oxidative stress or overproduction of proinflammatory cytokines clearly demonstrated in cellular models, *in vivo* studies showed that MnO_*x*_NPs induce brain damage through blood-brain barrier damage, alterations in neurotransmitter metabolism, and dysregulation of gut-brain axis.

## 4. Conclusions

**Fig. 2.**
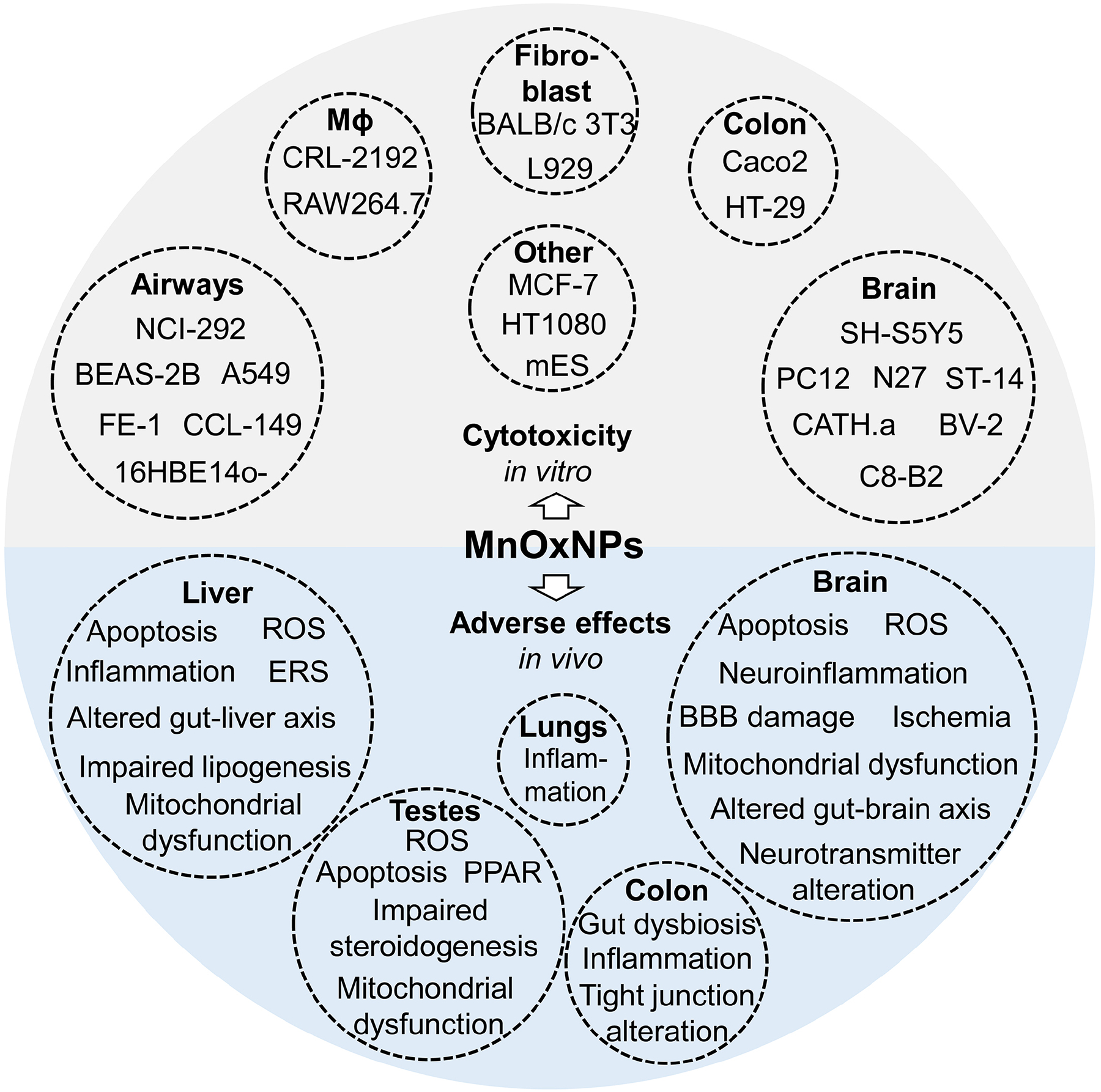
A summary of adverse effects of MnO_*x*_NPs in *in vitro* and *in vivo* models. The existing *in vitro* studies show that exposure to pristine MnOxNPs possesses cytotoxicity in brain, airway and lung, and colon cells, as well as macrophages, fibroblasts and other cell lines. The results from *in vivo* studies generally corroborate *in vitro* findings, demonstrating adverse effects of MnOxNPs exposure on brain, liver, testes, colon, and lungs.

Collectively, current evidence indicates that MnO_*x*_NPs, despite their biomedical applications as contrast or therapeutic agents, can exert toxic effects across multiple organs and tissues. Laboratory findings demonstrate hepatotoxicity, reproductive toxicity, as well as nephrotoxic and immunotoxic effects, while the brain emerges as a primary target of MnO_*x*_NPs accumulation (Fig. 2). Within the central nervous system, MnO_*x*_NPs trigger neuronal death through excessive ROS generation, oxidative stress, apoptosis, glial activation, and neuroinflammation. Specifically, MnO_*x*_NPs-induced mitochondrial dysfunction or direct prooxidant activity of the particles result in ROS overproduction which promotes cytochrome c release from the mitochondria and promote DNA oxidation, thus activating apoptosis. On the other hand, MnO_*x*_NPs-induced ROS generation, both directly and through MAPK signaling, promotes activation of redox-sensitive transcription factor NF-*κ*B and its nuclear translocation, leading to up-regulation of proinflammatory cytokines expression, resulting in neuroinflammation. *In vivo* studies generally corroborate cellular model studies, showing structural and functional impairments of the brain. Moreover, neurotoxicity is not only mediated by ROS and apoptosis but also involves blood–brain barrier disruption, impaired gut-brain axis, and dysregulation of neurotransmitter metabolism. It has also been shown that promotion of tau folding may also contribute to MnO_*x*_NPs neurotoxicity.

Despite being limited, the certain studies show that MnO_*x*_NPs possess comparable or even higher toxicity than soluble Mn^2+^ compounds, being indicative of the role of not only metal release, but also particle-specific effects in MnO_*x*_NPs-induced neurotoxicity. Furthermore, small-sized MnO_*x*_NPs possess more profound toxic effects compared to larger size NPs or microparticles. The existing data show that surface modifications like silica-coating or formation of nanocomposites of MnO_*x*_NPs reduces toxicity of the particles [17]. However, comparative analysis of toxic properties of pristine and modified MnO_*x*_NPs has not been performed. Although previous findings show that the shape of metal-containing NPs significantly affects their biological activity, data from comparative analysis of toxicity of MnO_*x*_NPs of various forms are lacking, while the majority of existing studies investigated the effects of spheric particles. Along with physical characteristics of the particles, chemical composition of MnO_*x*_NPs also affects its toxic properties. Although the data are scarce and controversial, it appears that Mn_2_O_3_NPs or Mn_3_O_4_NPs are more toxic than Mn_5_O_8_ and MnO_2_ *in vitro*. Finally, the way of particle synthesis also affects their toxicity, with green synthesis resulting in less toxic MnO_*x*_NPs compared to chemically synthesized particles.

Therefore, further studies are required to investigate the particular characteristics of MnO_*x*_NPs affecting its toxicity including size, shape, surface characteristics, and chemical composition. Specifically, comparative studies investigating the toxic effects of various MnO_*x*_NPs in a similar *in vivo* and *in vitro* models are highly required. Furthermore, more detailed studies investigating the molecular mechanisms of toxicity of MnO_*x*_NPs with distinct physicochemical characteristics are warranted to reveal the potential adverse effects of MnO_*x*_NPs exposure and ensure their safe application in biomedicine.

## Figures and Tables

**Table 1.** A summary of the key *in vitro* findings demonstrating adverse effects of MnOxNPs exposure on non-neuronal cells.

Cell model	Nanoparticles	Effects	Reference
NCI-292	Mn_2_O_3_, 20–100 nm size, 2.5–40 μg/cm^2^ for 24 h	Dose-dependent ↓ viability ↑ IL-8 secretion; IL-8, SOD2, HO-1 mRNA expression	[38]
BEAS-2B A549	Mn_2_O_3_, spherical, 82 nm size, 5–100 μg/mL for 24 h	Dose-dependent ↓ viability ↑ apoptosis/early necrosis	[42]
BEAS-2B RAW264.7	Mn_2_O_3_, 51.5 nm size, 50–200 μg/mL for 24 h	Dose-dependent ↓ viability (ATP and MTS assays) ↑ cell death; mitoROS; membrane permeability; mitochondrial membrane depolarization	[43]
Caco-2 A549 Balb/c 3T3	Mn_3_O_4_, <100 nm size, 6.25–100 μg/mL for 24 h	Dose-dependent ↓ viability EC_50_ (9-days): Caco-2—28.9 μg/mL; A549—47.1 μg/mL; Balb/c 3T3—49.9 μg/mL	[44]
A549 HT29	Mn_3_O_4_, spheric, 100 nm size, 1–10 mg/L for 24 h	Dose-dependent ↓ viability (A549) ↑ ROS production (A549, HT29)	[45]
A549	Mn_2_O_3_, spheric, 82 nm size, 20–100 μg/mL for 24–48 h	Dose-dependent ↓ viability, proliferation ↑ apoptosis	[46]
A549 MCF-7	Mn_3_O_4_, tetragonal, 36 nm size, 10–100 μg/mL for 24 h	Dose-dependent ↓ viability IC_50_ (A549) = 98 μg/mL; IC_50_ (MCF-7) = 25 μg/mL	[48]
FE1	MnO_2_, 40–60 nm size, vs MnO_2_MPs 5–10 μm size, 5–100 μg/mL for 2–4 h	Dose-dependent ↑ Percentage of DNA in the tail (MnO_2_NPs) ↑ micronucleus (↑ MPs, ↑ ↑ NPs)	[49]
CCL-149	Mn_3_O_4_, 30 nm size, 5–20 μg/mL for 24 h	↑ ROS production; GSSG level; caspase-3 activity; apoptosis (dose-dependent)	[50]
CRL-2192 CCL-149	Mn_3_O_4_, 20 nm size, 6 μg/cm^2^ cell surface for 4–24 h	↓ viability (CCL-149) ↓ viability (CRL-2192) ↓ NADPH oxidase activity, MCP1 (CRL-2192) ↑ ROS production (CRL-2192)	[51]
16HBE14o-	Mn_2_O_3_, 40–60 nm size, 13–500 mg/L for 48 h	IC_50_ = 33 mg/L	[52]
Raw 264.7	MnO_2_, spheric, 30–50 nm size, 6.25–100 μg/mL for 24 h	Dose-dependent ↓ viability; phagocytosis ↑ ROS production 12.5 μg/mL: ↑ TNF*α* and IL-1*β* mRNA; Nrf2 protein	[53]
Caco-2 A549 Balb/c 3T3	Mn_3_O_4_ 14.9 nm size, 3–100 μg/mL for 24 h	Dose-dependent ↓ viability ↑ ROS production IC_50_ (Caco-2) = 66.7; IC_50_ (A549) = 136.2; IC_50_ (Balb/c 3T3) = 195.0	[54]
Caco-2	MnO_2_, Mn_2_O_3_ and Mn_3_O_4_, spherical, 50 nm size, 10–500 μM for 24 h	Dose-dependent ↓ viability (Mn_3_O_4_ > Mn_2_O_3_ > MnO_2_) ↓ GSH (Mn_3_O_4_ > Mn_2_O_3_ > MnO_2_) Upon H_2_O_2_ exposure ↑ viability (Mn_2_O_3_ > MnO_2_ > Mn_3_O_4_)	[55]
MCF-7 HT1080	MnO_2_, nanoflakes, diameter—10–20 nm, length—80–120 nm, 25–100 μg/mL for 24 h	Dose-dependent ↓ viability; MMP; GSH level; SOD, CAT, GR activity ↑ ROS, TBARS, H_2_O_2_, mitoH_2_O_2_ 100 μg/mL: ↑ p53, Bax mRNA; cells in SubG_1_ ↓ Bcl2 mRNA; cells in S and G_2_/M Effects in HT1080 > MCF-7	[57]
HeLa L929	SiO_2_-coated MnO, 39.3 nm size, 10–200 μg/mL for 24 h	Dose-dependent ↓ viability; MMP; cells in G_1_ ↑ ROS, TBARS level; cells in G_2_/M ↑ apoptosis, caspase 3 activity Effects in L929 > HeLa	[58]
mES	Mn_3_O_4_, cubic, 20–180 nm and rods 8000 × 400 nm, 1–100 μg/mL for 24 h	Dose-dependent ↓ viability ↑ *Srxn1*, *Rtkn*, *Ddit3*, *Btg2* genes	[59]

*↑*, up-regulation; *↓*, down-regulation.

**Table 2.** A summary of the key *in vivo* studies demonstrating systemic toxicity of MnOxNPs.

Model	Exposure	Effects	Reference
Sprague-Dawley rats	Mn_3_O_4_, 15 nm size, 20 mg/kg i.p. for 120 days	Dose-dependent: ↑ liver Mn, ROS, MDA; ↑ apoptosis %, casp-3, Bax protein expression ↓ Bcl2 protein expression, GSH level, T-SOD activity ↑ *CYP1A2*, *Sult2a1*, *GRP78*, *Climp63* gene expression, CYP1A2 and Sult2a1 protein expression	[61]
*P. fulvidraco*	MnO_2_, 50 nm size, or MnSO_4_, 6 mg Mn/kg feed for 10 weeks	Vs MnSO_4_ ↑ serum ALT, AST ↑ liver SOD activity, GSSG/GSH ratio, lipid content ↑ *sod-1, sod-2, nrf2, il1β, il8, tnfa, bax, casp3, cycs, fas, acsl3, gpat3, agpat2, dgat1* mRNA expression ↓ *bcl2*, *il10* mRNA expression ↓ miR-92a, miR-92b-3p expression	[62]
*P. fulvidraco*	MnO_2_, 50 nm size, or MnSO_4_, 6 mg Mn/kg feed for 10 weeks	vs MnSO_4_: ↑ liver total lipids and TG content ↑ *mfn2*, *pgc1a* mRNA and protein expression ↓ *mief1* mRNA and protein expression ↓ miR-20a-5p expression	[63]
*P. f ulvidraco*	MnO_2_, 50 nm size, 20–80 mg/kg diet for 8 weeks	↑ liver Mn and lipid content ↑ serum ALT, AST ↑ *dmt1, fpn1, zip8, dnm1, cltc, il1β, tnfa, bax, casp3, cycs, keap1, hspbp1, hsp90a, hsf1, bnip3, bnip3Ia, march5, ulk1, beclin1, lc3b, p62, ctsb, tfeb* mRNA expression ↓ *zip14, cav1, dnm3, il10, bcl2, sod2, cat, gpx1* mRNA expression ↓ liver mtDNA, ATP content, CAT and SOD (low dose) activity, GSH/GSSG ↑ liver TBARS and SOD (high-dose), p-Hsf1S^326^, Hsf1, Atg7, LC3bII, BNIP3, mito-LC3bII, mito-BNIP3 protein expression	[64]
White feather chickens	Mn_2_O_3_, polygonal, 200 nm size, 900 mg Mn/kg diet for 3 weeks	↑ collagen fibers in liver, apoptosis, mitophagy ↑ serum TP, ALB, GLB, GGT, AST, LDH ↓ serum LDH; liver OCR; intestine villus height; intestine muscle layer width, goblet cell number ↑ liver cytc, APAF1, P53, caspase 8, Pink1, Parkin, ATG5, DRP1 mRNA expression ↓ liver Bcl2, P62, OPA1, MFN1, MFN2 mRNA expression ↓ liver caspase 9, P62 protein expression ↑ liver cytc, APAF1, P53, caspase 3, ATG5, MFF, FIS1 protein expression ↓ intestine MUC1, ZO-1 mRNA, occludin protein expression ↑ intestine TNF*α*, IL-1b mRNA, TNF*α* protein expression ↓ Sobs, Chao1, ACE, PD index in gut microbiota ↑ Firmicutes, Patescibacteria, *Lactobacillus*, *Pseudomonas*, *Ralstonia*, *Prauserella*, *Sedimini-bacterium*, *Burkholderia*-*Caballero*, *Paraburkholderia*, *Bradyrhizobium*, *Rubrobacter* ↓ *Bacteroidetes*, *Bacteroides*, *Bifidobacterium*	[65]
BALB c mice	MnO_2_, spherical, <40 nm size, 10 mg/kg interperitoneally for 50 days	Serum: ↑ AST, ALT, ALP, BUN, creatinine, bilirubin, glucose	[30]
Wistar rats	MnO_2_, 25–85 nm size, and MnO_2_MPs subcutaneous injection 100 μg/kg every 2 weeks for 14 weeks	↑ serum glucose, cholesterol (week 8), HDL-C (weeks 12 and 14) ↓ HDL-C (weeks 2, 4, 6, 8) ↓ LDL-C (weeks 6, 12, 14)	[66]
Sprague-Dawley rats	Mn_3_O_4_, spherical, 20 nm size, injected via tail vein every week 10 mg/kg/week for 60–120 days	↑ Mn content in serum and testes reduced thickness of the germinative layer and degeneration of seminiferous tubules ↓ sperm number, motility; serum testosterone, FSH; testes SOD ↑ sperm abnormality; testes TBARS, ROS ↓ fetus number, live pups% ↑ *Fabp1*, *Apoa2*, *Apoa3*, *Pck1*, *LOC100361547*, *Сур2c12*, *Cyp2c6v1*, *Ugt2b37 Gsta4*, *LOC102550391*, *Ugt2b37*, *Sult2a2*, *Gsta4*, *LOC102550391*, *Ugt2b37* gene expression	[67]
Sprague-Dawley rats	MnO_2_, lattice fringes shape, 50–100 nm size, subcutaneous injection with 100 mg/kg b.w. for 56 days	↓ BW, testis weight ↓ sperm count, motility, viability testicular degeneration, absence of germinal cells lining, exfoliation of immature germ cells, vascular congestion, edema ↑ total abnormality, detached head, bent tail frequency ↑ serum NOx, MDA ↓ serum TAC, GSH, FSH, LH, testosterone levels, CAT activity ↓ testes StAR, HSD-3*β*, CYP11A1 gene expression ↓ testes spermatogonia cells, primary spermatocyte cell, spermatid cell, Leydig cells ↓ seminiferous tubules diameter, epithelial height	[68]
Wistar rats	Mn_2_O_3_, 70 nm size, 100–400 ppm orally for 14 days	Dose-dependent: ↓ serum FSH, LH, testosterone levels ↓ testes spermatogonia cells, primary spermatocyte cell, spermatid cell, Leydig cells Interstitial edema of seminiferous tubules and vacuolar degeneration of epithelium	[69]
Wistar rats	MnO_2_, 25–85 nm size, subcutaneous injection with 100 mg/kg once a week for 4 weeks	↓ epididymal sperm count, motility ↓ spermatogonia, spermatocyte, seminiferous tubes diameter	[70]
Albino rats	Mn_3_O_4_, 18.4 nm size, intratracheal instillation with 0.25 mg per animal	↑ BALF total cellularity, neutrophilic leukocytes, neutrophil-to-alveolar macrophage ratio ↑ BALF amylase and LDH activity	[71]
C57 BL/6 mice	Mn_2_O_3_, 51.5 nm size, 20 μg by oropharyngeal installation for 40 h	↑ BALF neutrophil count, MCP-1 and IL-6 level	[43]
Kunming mice	Mn_3_O_4_, 6.0 to 10.5 nm size, via gavage 125–250 mg/kg BW for 20 days	↑ colon TBARS (high dose), AOC (low dose), IL-1*β* and IL-6 levels ↓ colon claudin-1 mRNA ↓ Chao1 index, number of species ↓ Verrucomicrobiiota, Bactieroidota, and *Firmicutes* C (both), *Spirochaetota* (low dose) ↑ *Proteobacteria*, Deferribacterota, *Desulfobacterota* I, and *Actinobacteria* (low dose), *Firmicutes*_A and *Firmicutes*_D (high dose) ↑ *Kineothrix*, *Alloprevotella*, OLB9, *Desulfovibrio*_R, *Muribaculum*, and UBA3282 (low dose), *OLB9*, *UBA3282*, *Treponema*_D, *CAG*-485, and *CAG*-873 (high dose) ↓ *Treponema*_D, *CAG*-485, and *CAG*-873 (low dose), *Kineothrix* and *Alloprevotella* (high dose)	[72]
Wistar rats	Mn_2_O_3_, 40–60 nm size, replacing MnCO_3_ in the diet at a dose of 65 mg Mn/kg diet for 16 weeks	Bone: fibrosis, steatosis	[74]

*↑*, up-regulation; *↓*, down-regulation.

**Table 3.** The key findings from *in vitro* studies on MnOxNPs neurotoxicity.

Cell model	Nanoparticles	Effects	Reference
PC12	Mn_3_O_4_, 25 nm size, 5–20 μg/mL for 24 h	Dose-dependent: ↓ viability, GSH level, SOD activity, mitochondrial membrane potential, dopamine content; Bcl2, caspase 3, caspase 9, DAT, DDC protein expression ↑ LDH release, MDA, apoptosis rate, cellular and mitochondrial Ca^2+^, ROS production; Bax, MCU, D2DR, TH protein expression	[33]
PC12	Mn, 40 nm size, irregularly shaped, 5–50 μg/mL for 24 h	Dose-dependent: ↓ viability, mitochondrial function ↑ ROS production ↓ DA, DOPAC (dose-dependent), HVA (high dose)	[34]
N27	Mn, 20 nm size, 25–400 μg/mL for 24 h	↓ viability (dose- and time-dependent), TH+ positive neuron number, TH+ cell neurite length ↑ total and mitochondrial ROS, caspase-3 and PKCδ activity, LCII and Beclin 1	[82]
PC12	Mn, 52 nm size, spherical shape, 10 μg/mL for 24 h	↓ Th, *α*-synuclein, Parkin gene expression	[83]
SH-SY5Y	MnO_2_, 40.6 nm size, 10–60 μg/mL for 24 and 48 h	Dose-dependent: ↓ viability, mitochondrial membrane potential, GSH level ↑ ROS, MDA, SOD, chromosome fragmentation, phosphatidylserine translocation, DNA damage	[84]
SH-SY5Y	Mn_2_O_3_, spherical, 30 nm size, 1–200 μg/mL for 24 h	Dose-dependent: ↓ viability; Bcl2 mRNA expression ↑ apoptosis and necrosis frequency, caspase-9 and caspase-3 activation, Bax mRNA expression IC_50_ = 88.45 μg/mL	[85]
ST-14	Mn_3_O_4_, 70–120 (A), 150–220 (B), and 200–350 (C) nm size, 1–10 μg/mL for 24 h	Dose-dependent: ↓ viability (A > B > C) ↑ LDH, ROS (A > B > C) ↑ NF-*κ*B activation (A)	[86]
BV2	MnO_2_, 2.5–10.0 μg/mL for 12 h	↑ IL-1*β* and TNF*α* expression; ROS production ↑ p38 MAPK activation	[87]
CATH.a and C8-B4 coculture	Mn, 65.8 nm size, irregular morphology, 10 or 50 μg/mL for 24 h	Dose-dependent: ↓ viability ↑ ROS production; IL-6 and TNF*α* mRNA expression ↑ miR-124–3p, miR-1–3p, miR-16–5p, let-7a-5p, and miR-155–5p expression; activation of pathways: molecular mechanism of cancer, role of Macs, Fibroblasts in RA, IL-6 signaling, NF-*κ*B signaling, IL-8 signaling	[88]

*↑*, up-regulation; *↓*, down-regulation.

**Table 4.** A summary of the key *in vivo* studies showing MnOxNPs neurotoxicity.

Model	Exposure	Effects	Reference
Wistar rats	MnO_2_, 23.2 nm size, intratracheal instillation with 2.63–5.26 mg Mn/kg b.w. 9 weeks	↓ Liver weight ↑ lungs weight ↑ time and count of local activity and immobility ↓ overall distance and time of ambulation ↑ latency of the evoked activity ↓ tail nerve conduction velocity	[89]
Wistar rats	MnO_2_, 9.14 (A), 42.36 (B), and 118.31 (C) nm size, intratracheal instillation with 3–6 mg/kg b.w. for 6 weeks	↓ body weight gain (A, B); thymus, liver (A, B), and spleen (A) weight ↑ lungs (all) and adrenals (A) weight ↑ time of immobility decreased and horizontal activity (all) ↓ slow frequency bands (delta, theta, alpha) ↑ fast bands (beta1, beta2, gamma) (A, B) ↓ latency of sensory evoked potentials (B, C)	[94]
Wistar rats	MnO_2_, 27.4 nm size, intratracheally instilled with 4 mg/kg b.w. for 6 weeks	↓ body weight gain ↑ TBARS in RBC and cortex ↑ frequencies ECoG in all three cortical areas (SS, VIS, AUD) ↓ evoked cortical and peripheral responses ↑ latency of the sensory evoked potentials (SS, VIS, AUD) ↓ tail nerve conduction velocity	[95]
Wistar rats	MnO_2_, 23 nm size, or MnCl_2_ in a dose of 2.53 mg Mn per rat intranasal instillation	↑ immobility; ↓ rearing (both) ↓ ambulation distance and time (MnCl_2_) ↑ time spent with local activity (MnCl_2_) ↓ slow frequency bands (delta, theta, alpha) (MnCl_2_) ↑ fast bands (beta1, beta2, gamma) (MnCl_2_) ↑ latency of the cortical evoked potentials (↑ nano, ↑ ↑ MnCl_2_) ↑ relative refractory period of the tail nerve (MnCl_2_)	[96]
Outbred white rats	Mn_3_O_4_, spherical, 18.4 nm size, intraperitoneally injected with 0.25–0.50 mg 3 times a week up to 18 injections	↓ Temporal summation of sub-threshold impulses, kidney mass (L), Number of head-dips into holes (H) ↓ RBC, lymphocytes; ↑ thrombocytes, monocytes, granulocytes count ↓ serum bilirubin, ALB, globulin, AST, uric acid, ceruloplasmin, thyroxin ↑ urinary UA and creatinine	[99]
C57 mice	MnO_2_, <100 nm size, vs MnO_2_-MPs single intravenous injection with 200 μL of 50 mg/kg	Vs MPs: ↓ complex II activity (brain, liver, kidney, heart, lung, ovary)	[101]
Wistar rats	MnO_*x*_, 15–29 nm size, inhalation with 0.029 mg/dm^3^ for 4 h	↓ complex IV activity (brain, lung) ↑ coordination impairment (low dose) ↓ response to auditory stimuli (low dose) neuronal and glial cell shrinkage and pyknosis, ischemia, and edema	[102]
Wistar rats	MnO_2_, 30 to 60 nm size, intraperitoneal injection with 50–100 μg/kg for 15 days	↑ immobility time (dose-dependent) ↓ sucrose consumption, hippocampal catecholamine level (dose-dependent) ↑ hippocampal ROS, MDA, apoptosis and necrosis rate	[105]
Sprague-Dawley rats	MnO_2_, 10 nm size, intracranial injection with 1 μL of 87 μg/μL	↑ abnormal behaviors (tremors, slow activity, sniffing, irritability and vertical tail) ↓ spatial learning; TH+ cells ↑ GFAP+, iNOS+ cells	[107]
Wistar rats	MnO_2_, 30–40 nm size, intraperitoneal injection with 10–20 mg/kg for 7 days	↑ blood-brain barrier leakage, neuronal injury, GFAP+ cells (dose-dependent) ↓ cerebral blood flow, Sensory motor functions (dose-dependent)	[35]
Sprague-Dawley rats	MnO_2_, 50 nm size, intratracheal injection with 200–400 mg/kg b.w. once a week for 3 months	↓ learning and memory, sensory ability of the hindlimbs Choroid plexus: epithelium nucleus disappearance, vacuolation, impaired cell junctions Hippocampus: edematous cells with karyopyknosis, irregular cell form and arrangement, cell connections loss ↑ Choroid plexus Brinp, Synpr, and Crmp1 mRNA expression	[36]
Wistar rats	Mn_2_O_3_, 40–60 nm size, 65 mg/kg replacing MnCO_3_for 12 weeks	Plasma: ↓ CRP level Jejunum: ↓ CRP and TNF*α* level; ↑ IL-6 level Brain: ↑ TNF*α* level and Cp activity	[108]
SD rats	MnO, 10 nm size, single intravenous injection with 10–40 mg Mn/kg b.w.	↑ GGT, ALP, ALT, AST activity; ↑ TC, LDL-C, crea, BUN level Brain metabolomics (both doses): ↑ lactate, creatine, Glu, cytidine, adenosine, Asp (early period) ↓ glucose, O-phosphoethanolamine, allantoin, taurine, PC, glycogen, Val, GPC, and Thr, Eth, NAD (early period) ↑ Phe, GABA, myo-inositol, uracil (all periods)	[110]
Wistar rats	Mn_2_O_3_, 40–60 nm size, 65 mg/kg replacing MnCO_3_ For 12 weeks	Caecum: ↓ Total SCFA, acetate, propionate, butyrate ↓ enzymatic activity of bacteria (*α*-Glucosidase, *β*-Glucosidase, *α*-Galactosidase, *β*-Galactosidase, *β*-Glucuronidase, *β*-Xylosidase, *α*-Arabinopyranosidase, *β*-Cellobiosidase, *β*-Mannosidase) Plasma: ↓ insulin, noradrenaline Intestine: ↓ histamine Brain: ↑ dopamine, noradrenaline; ↓ serotonine	[111]
Wistar rats	MnO_2_-NPs 42.63 nm size, at 30, 300, 1000 mg/kg b.w. or MnO_2_MPs at 1000 mg/kg single dose	↑ Blood leucocytes apoptosis (↑ MP, ↑ ↑ NPs) ↑ bone marrow cells micronucleus, chromosomal aberrations (↑ MP, ↑ ↑ NPs) Brain: ↓ AChE, Na/K-ATPase, Mg2+-ATPase, and Ca2+-ATPase RBC: ↓ AChE Liver, serum: ↑ ALT, AST Kidney: ↓ AST, ↑ ALT	[112]

*↑*, up-regulation; *↓*, down-regulation.

## References

[1] Morgan JJ. Manganese in natural waters and earth’s crust: its availability to organisms. Metal Ions in Biological Systems. 2000; 37: 1–34. 10693129

[2] Chen P, Bornhorst J, Aschner M. Manganese metabolism in humans. Frontiers in Bioscience (Landmark Edition). 2018; 23: 1655–1679. 10.2741/4665. 29293455

[3] Post JE. Manganese oxide minerals: crystal structures and economic and environmental significance. Proceedings of the National Academy of Sciences of the United States of America. 1999; 96: 3447–3454. 10.1073/pnas.96.7.3447. 10097056 PMC34287

[4] Vodyanitskii YN. Mineralogy and geochemistry of manganese: A review of publications. Eurasian Soil Science. 2009; 42: 1170–1178. 10.1134/S1064229309100123.

[5] Ding R, Chagnot M, Saeed S, Augustyn V. Nanostructured materials for electrochemical capacitors. In Reedijk J, Poeppelmeier KR, (eds.) Comprehensive Inorganic Chemistry III (pp. 225–240). 3rd edn. Elsevier: Amsterdam. 2023. 10.1016/B978-0-12-823144-9.00128-X.

[6] Haque S, Tripathy S, Patra CR. Manganese-based advanced nanoparticles for biomedical applications: future opportunity and challenges. Nanoscale. 2021; 13: 16405–16426. 10.1039/d1nr04964j. 34586121

[7] Manavalan RK, Enoch K, Volegov AS, Angusamy G, Nallasivam S. Review on medical applications of manganese oxide (Mn^2+^,Mn^3+^, and Mn^4+^) magnetic nanoparticles. Journal of Nanomaterials. 2024; 2024: 1073915. 10.1155/2024/1073915.

[8] Ishfaq A, Shahid M, Nawaz M, Ibrar D, Hussain S, Shahzad T, Remediation of wastewater by biosynthesized manganese oxide nanoparticles and its effects on development of wheat seedlings. Frontiers in Plant Science. 2023; 14: 1263813. 10.3389/fpls.2023.1263813. 38126015 PMC10731374

[9] Anegbe B, Ifijen IH. Recent advances in the application of manganese oxide nanoparticles for remediation of soil contaminated with organic pollutants. In TMS Annual Meeting & Exhibition (pp. 1358–1374). Springer Nature: Switzerland. 2024. 10.1007/978-3-031-50349-8_117.

[10] Rani N, Goswami B, Vats R, Jangra N, Bhukkal C, Ahlawat R. Multiphase manganese oxide nanoparticles suitable for optical and catalytic applications. Mater Today Proc. 2023 [In Press]. 10.1016/j.matpr.2023.03.158

[11] Rajagopalan K, Ramasubramanian B, Velusamy S, Ramakrishna S, Kannan AM, Kaliyannan M, Examining the economic and energy aspects of manganese oxide in Li-ion batteries. Materials Circular Economy. 2022; 4: 22. 10.1007/s42824-022-00064-4.

[12] Ghosh SK. Diversity in the Family of Manganese Oxides at the Nanoscale: From Fundamentals to Applications. ACS Omega. 2020; 5: 25493–25504. 10.1021/acsomega.0c03455. 33073076 PMC7557223

[13] Gowtham P, Girigoswami K, Pallavi P, Harini K, Gurubharath I, Girigoswami A. Alginate-Derivative Encapsulated Carbon Coated Manganese-Ferrite Nanodots for Multimodal Medical Imaging. Pharmaceutics. 2022; 14: 2550. 10.3390/pharmaceutics14122550. 36559045 PMC9782169

[14] Thirumalai A, Durgadevi P, Kiran V, Girigoswami K, Prabhu AD, Girigoswami A. Lipid functionalized silver-coated carbon dotcapped manganese ferrite as drug-free core-shell nanoparticles for multimodal imaging and therapy. ADMET & DMPK. 2025; 13: 2905. 10.5599/admet.2905. 41321378 PMC12662244

[15] Thirumalai A, Girigoswami K, Prabhu AD, Durgadevi P, Kiran V, Girigoswami A. 8-Anilino-1-naphthalenesulfonate-Conjugated Carbon-Coated Ferrite Nanodots for Fluoromagnetic Imaging, Smart Drug Delivery, and Biomolecular Sensing. Pharmaceutics. 2024; 16: 1378. 10.3390/pharmaceutics16111378. 39598502 PMC11597131

[16] Ding B, Zheng P, Ma P, Lin J. Manganese Oxide Nanomaterials: Synthesis, Properties, and Theranostic Applications. Advanced Materials (Deerfield Beach, Fla.). 2020; 32: e1905823. 10.1002/adma.201905823. 31990409

[17] Hsu BYW, Kirby G, Tan A, Seifalian AM, Li X, Wang J. Relaxivity and toxicological properties of manganese oxide nanoparticles for MRI applications. RSC Advances. 2016; 6: 45462–45474. 10.1039/C6RA04421B. 31156805 PMC6542684

[18] Sharmiladevi P, Girigoswami K, Haribabu V, Girigoswami A. Nano-enabled theranostics for cancer. Materials Advances. 2021; 2: 2876–2891. 10.1039/D1MA00069A.

[19] Khan S, Ansari AA, Khan AA, Abdulla M, Al-Obeed O, Ahmad R. In vitro evaluation of anticancer and biological activities of synthesized manganese oxide nanoparticles. MedChemComm. 2016; 7: 1647–1653. 10.1039/C6MD00219F.

[20] Yang G, Ji J, Liu Z. Multifunctional MnO_2_ nanoparticles for tumor microenvironment modulation and cancer therapy. Wiley Interdisciplinary Reviews. Nanomedicine and Nanobiotechnology. 2021; 13: e1720. 10.1002/wnan.1720. 33908171

[21] Xu Z, Qu A, Wang W, Lu M, Shi B, Chen C, Facet-Dependent Biodegradable Mn_3_O_4_ Nanoparticles for Ameliorating Parkinson’s Disease. Advanced Healthcare Materials. 2021; 10: e2101316. 10.1002/adhm.202101316. 34601811

[22] Singh N, Savanur MA, Srivastava S, D’Silva P, Mugesh G. A Redox Modulatory Mn_3_O_4_ Nanozyme with Multi-Enzyme Activity Provides Efficient Cytoprotection to Human Cells in a Parkinson’s Disease Model. Angewandte Chemie (International Ed. in English). 2017; 56: 14267–14271. 10.1002/anie.201708573. 28922532

[23] Gao W, Liu W, Dong X, Sun Y. Albumin-manganese dioxide nanocomposites: a potent inhibitor and ROS scavenger against Alzheimer’s *β*-amyloid fibrillogenesis and neuroinflammation. Journal of Materials Chemistry. B. 2023; 11: 10482–10496. 10.1039/d3tb01763j. 37909060

[24] Li LY, Park E, He C, Abbasi AZ, Henderson JT, Fraser PE, Evaluation of the biodistribution and preliminary safety profile of a novel brain-targeted manganese dioxide-based nanotheranostic system for Alzheimer’s disease. Nanotoxicology. 2024; 18: 315–334. 10.1080/17435390.2024.2361687. 38847611

[25] Li C, Zhao Z, Luo Y, Ning T, Liu P, Chen Q, Macrophage-Disguised Manganese Dioxide Nanoparticles for Neuroprotection by Reducing Oxidative Stress and Modulating Inflammatory Microenvironment in Acute Ischemic Stroke. Advanced Science (Weinheim, Baden-Wurttemberg, Germany). 2021; 8: e2101526. 10.1002/advs.202101526. 34436822 PMC8529435

[26] Sobańska Z, Roszak J, Kowalczyk K, Stępnik M. Applications and Biological Activity of Nanoparticles of Manganese and Manganese Oxides in In Vitro and In Vivo Models. Nanomaterials (Basel, Switzerland). 2021; 11: 1084. 10.3390/nano11051084. 33922170 PMC8145730

[27] Wang X, Yang X, Yi X, Min X, Jia Y. Rapid synthesis of manganese dioxide nanoparticles for enhanced biocompatibility and theranostic applications. RSC Advances. 2025; 15: 3060–3065. 10.1039/d4ra06995a. 39885857 PMC11780358

[28] Tinkov AA, Paoliello MMB, Mazilina AN, Skalny AV, Martins AC, Voskresenskaya ON, Molecular Targets of Manganese-Induced Neurotoxicity: A Five-Year Update. International Journal of Molecular Sciences. 2021; 22: 4646. 10.3390/ijms22094646. 33925013 PMC8124173

[29] Johnston LJ, Du X, Zborowski A, Kennedy DC. Characterization and Cellular Toxicity Studies of Commercial Manganese Oxide Nanoparticles. Nanomaterials (Basel, Switzerland). 2024; 14: 198. 10.3390/nano14020198. 38251162 PMC10821457

[30] Hafez AA, Naserzadeh P, Ashtari K, Mortazavian AM, Salimi A. Protection of manganese oxide nanoparticles-induced liver and kidney damage by vitamin D. Regulatory Toxicology and Pharmacology: RTP. 2018; 98: 240–244. 10.1016/j.yrtph.2018.08.005. 30102957

[31] Pardhiya S, Gaharwar US, Gautam R, Priyadarshini E, Nirala JP, Rajamani P. Cumulative effects of manganese nanoparticle and radiofrequency radiation in male Wistar rats. Drug and Chemical Toxicology. 2022; 45: 1395–1407. 10.1080/01480545.2020.1833905. 33111595

[32] Ikeda K, Tachibana T, Manome Y. The applications, neurotoxicity, and related mechanisms of manganese-containing nanoparticles. In Jiang X, Gao H, (eds.) Neurotoxicity of Nanomaterials and Nanomedicine (pp. 205–225). Academic Press: London. 2017. 10.1016/B978-0-12-804598-5.00009-X.

[33] Chen X, Wu G, Zhang Z, Ma X, Liu L. Neurotoxicity of Mn_3_O_4_ nanoparticles: Apoptosis and dopaminergic neurons damage pathway. Ecotoxicology and Environmental Safety. 2020; 188: 109909. 10.1016/j.ecoenv.2019.109909. 31740235

[34] Hussain SM, Javorina AK, Schrand AM, Duhart HM, Ali SF, Schlager JJ. The interaction of manganese nanoparticles with PC-12 cells induces dopamine depletion. Toxicological Sciences: an Official Journal of the Society of Toxicology. 2006; 92: 456–463. 10.1093/toxsci/kfl020. 16714391

[35] Sharma A, Feng L, Muresanu DF, Sahib S, Tian ZR, Lafuente JV, Manganese nanoparticles induce blood-brain barrier disruption, cerebral blood flow reduction, edema formation and brain pathology associated with cognitive and motor dysfunctions. Progress in Brain Research. 2021; 265: 385–406. 10.1016/bs.pbr.2021.06.015. 34560926

[36] Meng CY, Ma XY, Xu MY, Pei SF, Liu Y, Hao ZL, Transcriptomics-based investigation of manganese dioxide nanoparticle toxicity in rats’ choroid plexus. Scientific Reports. 2023; 13: 8510. 10.1038/s41598-023-35341-y. 37231062 PMC10213021

[37] Gotić M, Jurkin T, Musić S, Unfried K, Sydlik U, Bauer-Šegvić A. Microstructural characterizations of different Mn-oxide nanoparticles used as models in toxicity studies. Journal of Molecular Structure. 2013; 1044: 248–254. 10.1016/j.molstruc.2012.09.083.

[38] Delaval M, Wohlleben W, Landsiedel R, Baeza-Squiban A, Boland S. Assessment of the oxidative potential of nanoparticles by the cytochrome c assay: assay improvement and development of a high-throughput method to predict the toxicity of nanoparticles. Archives of Toxicology. 2017; 91: 163–177. 10.1007/s00204-016-1701-3. 27060086

[39] Luna-Velasco A, Field JA, Cobo-Curiel A, Sierra-Alvarez R. Inorganic nanoparticles enhance the production of reactive oxygen species (ROS) during the autoxidation of L-3,4-dihydroxyphenylalanine (L-dopa). Chemosphere. 2011; 85: 19–25. 10.1016/j.chemosphere.2011.06.053. 21737115

[40] Noventa S, Hacker C, Rowe D, Elgy C, Galloway T. Dissolution and bandgap paradigms for predicting the toxicity of metal oxide nanoparticles in the marine environment: an in vivo study with oyster embryos. Nanotoxicology. 2018; 12: 63–78. 10.1080/17435390.2017.1418920. 29262761

[41] McCarrick S, Wei Z, Moelijker N, Derr R, Persson KA, Hendriks G, High variability in toxicity of welding fume nanoparticles from stainless steel in lung cells and reporter cell lines: the role of particle reactivity and solubility. Nanotoxicology. 2019; 13: 1293–1309. 10.1080/17435390.2019.1650972. 31418618

[42] Chusuei CC, Wu CH, Mallavarapu S, Hou FYS, Hsu CM, Winiarz JG, Cytotoxicity in the age of nano: the role of fourth period transition metal oxide nanoparticle physicochemical properties. Chemico-biological Interactions. 2013; 206: 319–326. 10.1016/j.cbi.2013.09.020. 24120544

[43] Zhang H, Ji Z, Xia T, Meng H, Low-Kam C, Liu R, Use of metal oxide nanoparticle band gap to develop a predictive paradigm for oxidative stress and acute pulmonary inflammation. ACS Nano. 2012; 6: 4349–4368. 10.1021/nn3010087. 22502734 PMC4139054

[44] Titma T, Shimmo R, Siigur J, Kahru A. Toxicity of antimony, copper, cobalt, manganese, titanium and zinc oxide nanoparticles for the alveolar and intestinal epithelial barrier cells in vitro. Cytotechnology. 2016; 68: 2363–2377. 10.1007/s10616-016-0032-9. 27761772 PMC5101306

[45] Fernández-Pampín N, González Plaza JJ, García-Gómez A, Peña E, Rumbo C, Barros R, Toxicology assessment of manganese oxide nanomaterials with enhanced electrochemical properties using human in vitro models representing different exposure routes. Scientific Reports. 2022; 12: 20991. 10.1038/s41598-022-25483-w. 36471154 PMC9723098

[46] Tolliver LM, Holl NJ, Hou FYS, Lee HJ, Cambre MH, Huang YW. Differential Cytotoxicity Induced by Transition Metal Oxide Nanoparticles is a Function of Cell Killing and Suppression of Cell Proliferation. International Journal of Molecular Sciences. 2020; 21: 1731. 10.3390/ijms21051731. 32138333 PMC7084189

[47] Xia L, Park JH, Biggs K, Lee CG, Liao L, Shannahan JH. Compositional variations in metal nanoparticle components of welding fumes impact lung epithelial cell toxicity. Journal of Toxicology and Environmental Health. Part a. 2023; 86: 735–757. 10.1080/15287394.2023.2238209. 37485994

[48] Shaik MR, Syed R, Adil SF, Kuniyil M, Khan M, Alqahtani MS, Mn_3_O_4_ nanoparticles: Synthesis, characterization and their antimicrobial and anticancer activity against A549 and MCF-7 cell lines. Saudi Journal of Biological Sciences. 2021; 28: 1196–1202. 10.1016/j.sjbs.2020.11.087. 33613047 PMC7878830

[49] Solorio-Rodriguez SA, Wu D, Boyadzhiev A, Christ C, Williams A, Halappanavar S. A Systematic Genotoxicity Assessment of a Suite of Metal Oxide Nanoparticles Reveals Their DNA Damaging and Clastogenic Potential. Nanomaterials (Basel, Switzerland). 2024; 14: 743. 10.3390/nano14090743. 38727337 PMC11085103

[50] Frick R, Müller-Edenborn B, Schlicker A, Rothen-Rutishauser B, Raemy DO, Günther D, Comparison of manganese oxide nanoparticles and manganese sulfate with regard to oxidative stress, uptake and apoptosis in alveolar epithelial cells. Toxicology Letters. 2011; 205: 163–172. 10.1016/j.toxlet.2011.05.1037. 21669262

[51] Urner M, Schlicker A, Z’graggen BR, Stepuk A, Booy C, Buehler KP, Inflammatory response of lung macrophages and epithelial cells after exposure to redox active nanoparticles: effect of solubility and antioxidant treatment. Environmental Science & Technology. 2014; 48: 13960–13968. 10.1021/es504011m. 25343230

[52] Otero-González L, Sierra-Alvarez R, Boitano S, Field JA. Application and validation of an impedance-based real time cell analyzer to measure the toxicity of nanoparticles impacting human bronchial epithelial cells. Environmental Science & Technology. 2012; 46: 10271–10278. 10.1021/es301599f. 22916708

[53] Alsaleh NB, Aljarbou AM, Assal ME, Assiri MA, Almutairi MM, As Sobeai HM, Synthesis, characterization, and toxicity assessment of zinc oxide-doped manganese oxide nanoparticles in a macrophage model. Pharmaceuticals. 2024; 17: 168. 10.3390/ph17020168. 38399383 PMC10892842

[54] Ivask A, Titma T, Visnapuu M, Vija H, Kakinen A, Sihtmae M, Toxicity of 11 Metal Oxide Nanoparticles to Three Mammalian Cell Types In Vitro. Current Topics in Medicinal Chemistry. 2015; 15: 1914–1929. 10.2174/1568026615666150506150109. 25961521

[55] Jiang X, Gray P, Patel M, Zheng J, Yin JJ. Crossover between anti- and pro-oxidant activities of different manganese oxide nanoparticles and their biological implications. Journal of Materials Chemistry. B. 2020; 8: 1191–1201. 10.1039/c9tb02524c. 31967629

[56] Tootoonchi MH, Hashempour M, Blackwelder PL, Fraker CA. Manganese oxide particles as cytoprotective, oxygen generating agents. Acta Biomaterialia. 2017; 59: 327–337. 10.1016/j.actbio.2017.07.006. 28688986

[57] Alhadlaq HA, Akhtar MJ, Ahamed M. Different cytotoxic and apoptotic responses of MCF-7 and HT1080 cells to MnO_2_ nanoparticles are based on similar mode of action. Toxicology. 2019; 411: 71–80. 10.1016/j.tox.2018.10.023. 30395893

[58] Yu C, Zhou Z, Wang J, Sun J, Liu W, Sun Y, In depth analysis of apoptosis induced by silica coated manganese oxide nanoparticles in vitro. Journal of Hazardous Materials. 2015; 283: 519–528. 10.1016/j.jhazmat.2014.09.060. 25464291

[59] McCarrick S, Cappellini F, Kessler A, Moelijker N, Derr R, Hedberg J, ToxTracker Reporter Cell Lines as a Tool for Mechanism-Based (geno)Toxicity Screening of Nanoparticles-Metals, Oxides and Quantum Dots. Nanomaterials (Basel, Switzerland). 2020; 10: 110. 10.3390/nano10010110. 31935871 PMC7023144

[60] Lu H, Zhang X, Khan SA, Li W, Wan L. Biogenic Synthesis of MnO_2_ Nanoparticles With Leaf Extract of *Viola betonicifolia* for Enhanced Antioxidant, Antimicrobial, Cytotoxic, and Biocompatible Applications. Frontiers in Microbiology. 2021; 12: 761084. 10.3389/fmicb.2021.761084. 34790185 PMC8591690

[61] Yue Z, Zhang X, Yu Q, Liu L, Zhou X. Cytochrome P450-dependent reactive oxygen species (ROS) production contributes to Mn_3_O_4_ nanoparticle-caused liver injury. RSC Advances. 2018; 8: 37307–37314. 10.1039/c8ra05633a. 35557821 PMC9089396

[62] Zhao T, Zheng H, Xu JJ, Xu YC, Liu LL, Luo Z. MnO_2_ nanoparticles and MnSO_4_ differentially affected hepatic lipid metabolism through miR-92a/acsl3-dependent de novo lipogenesis in yellow catfish Pelteobagrusfulvidraco. Environmental Pollution (Barking, Essex: 1987). 2023; 336: 122416. 10.1016/j.envpol.2023.122416. 37598932

[63] Zhao T, Tan XY, Pantopoulos K, Xu JJ, Zheng H, Xu YC, miR-20a-5p targeting mfn2-mediated mitochondria-lipid droplet contacts regulated differential changes in hepatic lipid metabolism induced by two Mn sources in yellow catfish. Journal of Hazardous Materials. 2024; 462: 132749. 10.1016/j.jhazmat.2023.132749. 37871441

[64] Zhao T, Zheng H, Xu JJ, Pantopoulos K, Xu YC, Liu LL, MnO_2_ nanoparticles trigger hepatic lipotoxicity and mitophagy via mtROS-dependent Hsf1^*Ser*326^ phosphorylation. Free Radical Biology & Medicine. 2024; 210: 390–405. 10.1016/j.freeradbiomed.2023.11.037. 38048852

[65] Li Y, Yi J, Liu K, Liu X, Yangzom C, Pan J, Mn_2_O_3_ NPs-induced liver injury is potentially associated with gut microbiota dysbiosis in broiler chicken. Food and Chemical Toxicology: an International Journal Published for the British Industrial Biological Research Association. 2025; 202: 115487. 10.1016/j.fct.2025.115487. 40288515

[66] Mousavi Z, PhD, Hassanpourezatti M, PhD, Najafizadeh P, PhD, Rezagholian S, Ms, Rhamanifar MS, PhD, Nosrati N, Ms. Effects of Subcutaneous Injection MnO_2_ Micro- and Nanoparticles on Blood Glucose Level and Lipid Profile in Rat. Iranian Journal of Medical Sciences. 2016; 41: 518–524. 27853332 PMC5106567

[67] Zhang X, Yue Z, Zhang H, Liu L, Zhou X. Repeated administrations of Mn_3_O_4_ nanoparticles cause testis damage and fertility decrease through PPAR-signaling pathway. Nanotoxicology. 2020; 14: 326–340. 10.1080/17435390.2019.1695976. 31909642

[68] Ramadan HM, Taha NA, Youssef AM, Morsi AS. Rosemary essenitial oil counters MnO_2_ nanoparticle-induced fertility deficits in rats via antioxidant mechanisms and upregulation of StAR signalling. Scientific Reports. 2025; 15: 20201. 10.1038/s41598-025-06345-7. 40542149 PMC12181236

[69] Negahdary M, Arefian Z, Dastjerdi HA, Ajdary M. Toxic effects of Mn2O3 nanoparticles on rat testis and sex hormone. Journal of Natural Science, Biology, and Medicine. 2015; 6: 335–339. 10.4103/0976-9668.159998. 26283824 PMC4518404

[70] Yousefalizadegan N, Mousavi Z, Rastegar T, Razavi Y, Najafizadeh P. Reproductive toxicity of manganese dioxide in forms of micro- and nanoparticles in male rats. International Journal of Reproductive Biomedicine. 2018; 17: 361–370. 10.18502/ijrm.v17i5.4603. 31435611 PMC6653491

[71] Sutunkova MP, Klinova SV, Ryabova YV, Tazhigulova AV, Minigalieva IA, Shabardina LV, Comparative Evaluation of the Cytotoxic Effects of Metal Oxide and Metalloid Oxide Nanoparticles: An Experimental Study. International Journal of Molecular Sciences. 2023; 24: 8383. 10.3390/ijms24098383. 37176090 PMC10178919

[72] Zhang B, Yang L, Wu Z, Wang X, Zhao X, Zhang W, Effect of oral Mn-based nanozymes Mn_3_O_4_ NPs on morphological, antioxidation, mucosa, and fecal microbial community in mice colons. Food and Chemical Toxicology: an International Journal Published for the British Industrial Biological Research Association. 2025; 197: 115313. 10.1016/j.fct.2025.115313. 39923832

[73] Kot Y, Prokopiuk V, Klochkov V, Tryfonyuk L, Maksimchuk P, Aslanov A, Mn_3_O_4_ Nanocrystal-Induced Eryptosis Features Ca^2+^ Overload, ROS and RNS Accumulation, Calpain Activation, Recruitment of Caspases, and Changes in the Lipid Order of Cell Membranes. International Journal of Molecular Sciences. 2025; 26: 3284. 10.3390/ijms26073284. 40244142 PMC11989249

[74] Cholewińska E, Dworzański W, Juśkiewicz J, Listos P, Ognik K. Consequences of Dietary Manganese Deficiency or Mn_2_O_3_ Nanoparticles Supplementation on Rat Manganese Biodistribution and Femur Morphology. Nutrients. 2025; 17: 3184. 10.3390/nu17193184. 41097261 PMC12526447

[75] Sabaghi S, Razmyar J, Heidarpour M. Effects of nano-manganese on humoral immune response and oxidative stress in broilers. Veterinary Research Forum: an International Quarterly Journal. 2021; 12: 487–491. 10.30466/vrf.2020.114233.2716. 35529808 PMC9010834

[76] Jankowski J, Ognik K, Stępniowska A, Zduńczyk Z, Kozłowski K. The effect of manganese nanoparticles on apoptosis and on redox and immune status in the tissues of young turkeys. PloS One. 2018; 13: e0201487. 10.1371/journal.pone.0201487. 30063726 PMC6067725

[77] Haque S, Kotcherlakota R, Bhamidipati P, Muralidharan K, Sreedhar B, Amanchy R, Smartly engineered casein manganese oxide nanobiomaterials and its potential therapeutic angiogenesis applications for wound healing and limb ischemia. Advanced Therapeutics. 2023; 6: 2300142. 10.1002/adtp.202300142.

[78] Haque S, Tripathy S, Chandra Y, Muralidharan K, Patra CR. Toxicity study of pro-angiogenic casein manganese oxide nanoparticles: an *in vitro* and *in vivo* approach. Nanotoxicology. 2023; 17: 604–627. 10.1080/17435390.2023.2291788. 38105710

[79] Harini K, Girigoswami K, Anand AV, Pallavi P, Gowtham P, Elboughdiri N, Nano-mediated strategies for metal ioninduced neurodegenerative disorders: focus on Alzheimer’s and Parkinson’s diseases. Current Pharmacology Reports. 2022; 8: 450–463. 10.1007/s40495-022-00307-7.

[80] Huang Y, Ruan Y, Ma Y, Chen D, Zhang T, Fan S, Immunomodulatory activity of manganese dioxide nanoparticles: Promising for novel vaccines and immunotherapeutics. Frontiers in Immunology. 2023; 14: 1128840. 10.3389/fimmu.2023.1128840. 36926351 PMC10011163

[81] Ullah AA, Akter M, Tareq ARM, Kibria AF, Firoz SH. Deciphering MnO_*x*_ nanoparticles: A comprehensive analysis reveals Mn_2_O_3_ as the most cytotoxic variant in cellular systems. Materials Letters. 2024; 355: 135520. 10.1016/j.matlet.2023.135520.

[82] Afeseh Ngwa H, Kanthasamy A, Gu Y, Fang N, Anantharam V, Kanthasamy AG. Manganese nanoparticle activates mitochondrial dependent apoptotic signaling and autophagy in dopaminergic neuronal cells. Toxicology and Applied Pharmacology. 2011; 256: 227–240. 10.1016/j.taap.2011.07.018. 21856324 PMC3205240

[83] Wang J, Rahman MF, Duhart HM, Newport GD, Patterson TA, Murdock RC, Expression changes of dopaminergic systemrelated genes in PC12 cells induced by manganese, silver, or copper nanoparticles. Neurotoxicology. 2009; 30: 926–933. 10.1016/j.neuro.2009.09.005. 19781568

[84] Alarifi S, Ali D, Alkahtani S. Oxidative Stress-Induced DNA Damage by Manganese Dioxide Nanoparticles in Human Neuronal Cells. BioMed Research International. 2017; 2017: 5478790. 10.1155/2017/5478790. 28596964 PMC5449756

[85] Mehdizadeh P, Fesharaki SSH, Nouri M, Ale-Ebrahim M, Akhtari K, Shahpasand K, Tau folding and cytotoxicity of neuroblastoma cells in the presence of manganese oxide nanoparticles: Biophysical, molecular dynamics, cellular, and molecular studies. International Journal of Biological Macromolecules. 2019; 125: 674–682. 10.1016/j.ijbiomac.2018.11.191. 30468808

[86] Stefanescu DM, Khoshnan A, Patterson PH, Hering JG. Neurotoxicity of manganese oxide nanomaterials. Journal of Nanoparticle Research. 2009; 11: 1957–1969. 10.1007/s11051-008-9554-1.

[87] Sun X, Qin X, Liang G, Chang X, Zhu H, Zhang J, Manganese dioxide nanoparticles provoke inflammatory damage in BV2 microglial cells via increasing reactive oxygen species to activate the p38 MAPK pathway. Toxicology and Industrial Health. 2024; 40: 244–253. 10.1177/07482337241242508. 38518383

[88] Grogg MW, Braydich-Stolle LK, Maurer-Gardner EI, Hill NT, Sakaram S, Kadakia MP, Modulation of miRNA-155 alters manganese nanoparticle-induced inflammatory response. Toxicology Research. 2016; 5: 1733–1743. 10.1039/c6tx00208k. 30090472 PMC6062203

[89] Takács SZ, Szabó A, Oszlánczi G, Pusztai P, Sápi A, Kónya Z, Repeated simultaneous cortical electrophysiological and behavioral recording in rats exposed to manganese-containing nanoparticles. Acta Biologica Hungarica. 2012; 63: 426–440. 10.1556/ABiol.63.2012.4.2. 23134600

[90] Sárközi L, Horváth E, Kónya Z, Kiricsi I, Szalay B, Vezér T, Subacute intratracheal exposure of rats to manganese nanoparticles: behavioral, electrophysiological, and general toxicological effects. Inhalation Toxicology. 2009; 21 Suppl 1: 83–91. 10.1080/08958370902939406. 19558238

[91] Oszlánczi G, Vezér T, Sárközi L, Horváth E, Kónya Z, Papp A. Functional neurotoxicity of Mn-containing nanoparticles in rats. Ecotoxicology and Environmental Safety. 2010; 73: 2004–2009. 10.1016/j.ecoenv.2010.09.002. 20863568

[92] Elder A, Gelein R, Silva V, Feikert T, Opanashuk L, Carter J, Translocation of inhaled ultrafine manganese oxide particles to the central nervous system. Environmental Health Perspectives. 2006; 114: 1172–1178. 10.1289/ehp.9030. 16882521 PMC1552007

[93] Romashchenko AV, Sharapova MB, Morozova KN, Kiseleva EV, Kuper KE, Petrovskii DV. The role of olfactory transport in the penetration of manganese oxide nanoparticles from blood into the brain. Vavilov J Genet Breeding. 2019; 23: 482–488. 10.18699/VJ19.517.

[94] Máté Z, Horváth E, Kozma G, Simon T, Kónya Z, Paulik E, Size-Dependent Toxicity Differences of Intratracheally Instilled Manganese Oxide Nanoparticles: Conclusions of a Subacute Animal Experiment. Biological Trace Element Research. 2016; 171: 156–166. 10.1007/s12011-015-0508-z. 26384687

[95] Sárközi K, Papp A, Horváth E, Máté Z, Hermesz E, Kozma G, Protective effect of green tea against neuro-functional alterations in rats treated with MnO_2_ nanoparticles. Journal of the Science of Food and Agriculture. 2017; 97: 1717–1724. 10.1002/jsfa.7919. 27435261

[96] Oszlánczi G, Vezér T, Sárközi L, Horváth E, Szabó A, Horváth E, Metal deposition and functional neurotoxicity in rats after 3–6 weeks nasal exposure by two physicochemical forms of manganese. Environmental Toxicology and Pharmacology. 2010; 30: 121–126. 10.1016/j.etap.2010.04.006. 21787641

[97] Horváth E, Máté Z, Takács S, Pusztai P, Sápi A, Kónya Z, General and electrophysiological toxic effects of manganese in rats following subacute administration in dissolved and nanoparticle form. TheScientificWorldJournal. 2012; 2012: 520632. 10.1100/2012/520632. PMC3361337 22654621

[98] Sárközi K, Nagy V, Papp A, Csákiné Tombácz E, Szabó A. Effects of three natural antioxidants on the general and nervous system toxicity of manganese nanoparticles in rats. Central European Journal of Occupational and Environmental Medicine. 2013; 19: 31–42.

[99] Katsnelson BA, Minigaliyeva IA, Panov VG, Privalova LI, Varaksin AN, Gurvich VB, Some patterns of metallic nanoparticles’ combined subchronic toxicity as exemplified by a combination of nickel and manganese oxide nanoparticles. Food and Chemical Toxicology: an International Journal Published for the British Industrial Biological Research Association. 2015; 86: 351–364. 10.1016/j.fct.2015.11.012. 26607108

[100] Minigalieva IA, Katsnelson BA, Privalova LI, Sutunkova MP, Gurvich VB, Shur VY, Attenuation of Combined Nickel(II) Oxide and Manganese(II, III) Oxide Nanoparticles’ Adverse Effects with a Complex of Bioprotectors. International Journal of Molecular Sciences. 2015; 16: 22555–22583. 10.3390/ijms160922555. 26393577 PMC4613324

[101] Ashrafi Hafez A, Naserzadeh P, Mortazavian AM, Mehravi B, Ashtari K, Seydi E, Comparison of the effects of MnO_2_-NPs and MnO_2_-MPs on mitochondrial complexes in different organs. Toxicology Mechanisms and Methods. 2019; 29: 86–94. 10.1080/15376516.2018.1512693. 30132356

[102] Zaitseva NV, Zemlyanova MA, Zvezdin VN, Akafieva TI, Saenko EV. Acute inhalation toxicity of manganese oxide nanoparticles. Nanotechnologies in Russia. 2015; 10: 468–474. 10.1134/S1995078015030180.

[103] Zvezdin VN, Zemlyanova MA, Akafieva TI. Inhalation toxicity of nanodispersed manganese oxide aerosol. Meditsina Truda I Promyshlennaia Ekologiia. 2015; 13–16. 27024922

[104] Zaitseva NV, Zemlyanova MA. Toxicologic characteristics of nanodisperse manganese oxide: physical-chemical properties, biological accumulation, and morphological-functional properties at various exposure types. In Nduka JK, Rashed MN (eds.) Heavy Metal Toxicity in Public Health. IntechOpen: Rijeka. 2019. 10.5772/intechopen.83499.

[105] Sadeghi L, Babadi VY, Tanwir F. Manganese dioxide nanoparticle induces Parkinson like neurobehavioral abnormalities in rats. Bratislavske Lekarske Listy. 2018; 119: 379–384. 10.4149/BLL_2018_070. 29947239

[106] Máté Z, Horváth E, Papp A, Kovács K, Tombácz E, Nesztor D, Neurotoxic effects of subchronic intratracheal Mn nanoparticle exposure alone and in combination with other welding fume metals in rats. Inhalation Toxicology. 2017; 29: 227–238. 10.1080/08958378.2017.1350218. 28722486

[107] Li T, Shi T, Li X, Zeng S, Yin L, Pu Y. Effects of Nano-MnO2 on dopaminergic neurons and the spatial learning capability of rats. International Journal of Environmental Research and Public Health. 2014; 11: 7918–7930. 10.3390/ijerph110807918. 25101772 PMC4143840

[108] Różaniecka-Zwolińska K, Cholewińska E, Fotschki B, Juśkiewicz J, Ognik K. Manganese deficiency or dietary manganese(III) oxide nanoparticle supplementation: consequences for hematology, and intestinal and brain immunity in rats. Frontiers in Immunology. 2025; 16: 1528770. 10.3389/fimmu.2025.1528770. 40264758 PMC12011558

[109] Zaĭtseva NV, Zemlianova MA, Zvezdin VN, Saenko EV, Tarantin AV, Makhmudov RR, Hygienic and toxicological safety assessment of nano- and micro-dispersed manganese oxide (III, IV). Voprosy Pitaniia. 2012; 81: 13–19. 23461167

[110] Li J, Zhao Z, Feng J, Gao J, Chen Z. Understanding the metabolic fate and assessing the biosafety of MnO nanoparticles by metabonomic analysis. Nanotechnology. 2013; 24: 455102. 10.1088/0957-4484/24/45/455102. 24145610

[111] Sołek P, Różaniecka K, Juśkiewicz J, Fotschki B, Stępniowska A, Ognik K. Consequences of Dietary Manganese-Based Nanoparticles Supplementation or Deficiency on Systemic Health and Gut Metabolic Dynamics in Rats. Nanotechnology, Science and Applications. 2025; 18: 19–34. 10.2147/NSA.S494533. 39981122 PMC11840336

[112] Singh SP, Kumari M, Kumari SI, Rahman MF, Mahboob M, Grover P. Toxicity assessment of manganese oxide micro and nanoparticles in Wistar rats after 28 days of repeated oral exposure. Journal of Applied Toxicology: JAT. 2013; 33: 1165–1179. 10.1002/jat.2887. 23702825

[113] Kumar N, Thorat ST, Reddy KS. Multi biomarker approach to assess manganese and manganese nanoparticles toxicity in Pangasianodon hypophthalmus. Scientific Reports. 2023; 13: 8505. 10.1038/s41598-023-35787-0. 37231182 PMC10213040

[114] Illarionova NB, Morozova KN, Petrovskii DV, Sharapova MB, Romashchenko AV, Troitskii SY, ‘Trojan-Horse’ stress-granule formation mediated by manganese oxide nanoparticles. Nanotoxicology. 2020; 14: 1432–1444. 10.1080/17435390.2020.1856433. 33320703

